# Elp3 uses a conserved molecular tunnel to transport acetate between distant active sites and catalyze tRNA wobble base modification

**DOI:** 10.1038/s41467-026-73699-5

**Published:** 2026-06-03

**Authors:** Evan P. Geissler, Youmna Moawad, Paige N. Roehling, Cassidy Driscoll, Katherine Martin, Papa Nii Asare-Okai, Jeffrey S. Mugridge

**Affiliations:** Department of Chemistry & Biochemistry, University of Delaware, Newark, DE 19716 USA

**Keywords:** RNA, Enzyme mechanisms, Metalloproteins, RNA-binding proteins

## Abstract

The radical SAM enzyme Elp3 and eukaryotic Elongator complex catalyze formation of a key intermediate transfer RNA (tRNA) modification, 5-carboxymethyluridine (cm^5^U), in the anticodons of tRNAs across all domains of life. cm^5^U-derived modifications are important for fine tuning codon-anticodon interactions and efficient protein translation, and defects in this modification are linked to development of neurodegenerative disease in humans. Here we reconstitute tRNA modification activity with a model Elp3 enzyme and combine structural analyses, enzymology, and isotope incorporation experiments to show Elp3 harbors a conserved molecular tunnel that shuttles free acetate molecules from the acetyl-CoA binding domain to the radical SAM active site over 20 Å away, where acetate undergoes radical-mediated reaction and addition to tRNA U34. Our model explains how Elp3 bridges a large distance between active sites to catalyze tRNA carboxymethylation and illustrates a unique mechanism for intermediate transport in radical SAM enzymes.

## Abstract (web summary)

tRNA modifications are essential for accurate translation, but how Elp3 catalyses them across distant active sites was unclear. Here, the authors show that Elp3 uses a conserved molecular tunnel to transport acetate for radical-mediated tRNA carboxymethylation

## Introduction

Organisms across all domains of life decorate their transfer RNA (tRNA) molecules with a wide variety of chemical modifications that finely tune protein translation and are critical for protein homeostasis and cellular health^1–4^. Over 100 distinct tRNA nucleobase modifications have been identified to date^5^, with an average of 13 modifications per tRNA found in human cells^6^. 5-carboxymethyluridine (cm^5^U)-derived modifications (Supplementary Fig. 1) are found on over 50% of wobble uridines at position 34 in eukaryotic tRNAs^7^ and critically modulate the structure and function of the tRNA anticodon loop^8^. This family of cm^5^U34-derived tRNA modifications facilitate base pair recognition between the mRNA codon and tRNA anticodon in the ribosome, ensuring efficient and accurate decoding and protein translation^9–11^. Defects in the cm^5^U34-derived modification pathways lead to detrimental cellular phenotypes and disease outcomes. For example, proper levels of 5-methoxycarbonylmethyluridine 34 (mcm^5^U34)-modified tRNAs are required for effective translation of multiple DNA damage repair proteins and correct progression through the cell cycle in yeast^12–14^, while reduction of mcm^5^U34 levels impairs selenocysteine protein expression and increases cellular sensitivity to reactive oxygen species in mammals^15–17^. Loss of the related 5-methoxycarbonylmethyl-2-thiouridine (mcm^5^s^2^U) modification leads to frameshift errors during translation^18^ and dysregulation of metabolic^19^ and protein homeostasis^20–22^ in yeast, and is strongly linked to the development of neurodegenerative diseases and neurodevelopmental disorders, including familial dysautonomia, in humans^23^.

In eukaryotes, the multiprotein Elongator complex is responsible for installing cm^5^U34 on tRNA and additional tRNA-modifying enzymes subsequently act on cm^5^U34 to generate the family of cm^5^U34-derived modifications described above (Supplementary Fig. 1)^15,24–28^. The Elongator complex is composed of two copies each of Elongator proteins 1-6 (Elp1-6), where Elp3 is the enzymatic core of the complex that carries out the tRNA modification reaction and the other Elp proteins are thought to act as adaptor or scaffolding proteins that facilitate tRNA binding and release^29,30^. In most archaea and numerous bacteria, only the catalytic subunit Elp3 is conserved and so this enzyme likely performs the tRNA modification reaction alone in those organisms where cm^5^U is formed^31^. Structures of Elp3 have been determined from all three domains of life, revealing a highly conserved architecture consisting of a radical *S*-adenosylmethionine (rSAM) domain that binds a [4Fe-4S] cluster and cofactor SAM, and a lysine acetyltransferase (KAT) domain that binds acetyl-CoA (AcCoA) (Supplementary Fig. 2)^32–35^. Mutations in Elp3 and Elongator disrupt protein folding and stress responses^36,37^, and are closely associated with the development and progression of a variety of human diseases, including various cancers^38–41^ and neurodevelopmental^42,43^ and neurodegenerative disorders^11,44^ such as familial dysautonomia^45^ and amyotrophic lateral sclerosis (ALS)^46,47^.

Elp3 is thought to use a multistep mechanism in which tRNA binding triggers canonical rSAM chemistry at the [4Fe-4S] cluster to reductively cleave SAM and generate a reactive 5′-deoxyadenosyl radical (5′-dA·), which subsequently reacts with AcCoA or the acetyl group from AcCoA, to ultimately generate the cm^5^U34 modification on tRNA^31^. However, recent structures of Elp3 and the Elongator complex with bound tRNA all show that the [4Fe-4S] active site on the rSAM domain and the AcCoA binding site on the KAT domain are separated by over 20 Å^30,32–35^. This raises critical questions about how SAM (or 5′-dA·) and AcCoA cofactors come together in the Elp3 rSAM active site to react with tRNA and form the key cm^5^U wobble base modification. To date, there has only been a single report from 2014 in which any Elp3- or Elongator-mediated tRNA modifying activity has been detected with recombinant enzyme(s) in vitro^31^; this challenge has so far prevented careful enzymological investigation and clear understanding of how Elp3 and Elongator modify tRNA.

Here we successfully reconstitute the in vitro cm^5^U34 tRNA modification reaction for the first time in over a decade using a model, recombinant archaeal Elp3 and combine structural analysis with enzymology and isotope incorporation experiments to propose a new mechanism for Elp3-mediated tRNA modification. We show that Elp3-tRNA complexes contain a conserved, enclosed molecular tunnel connecting the distant rSAM and KAT sites, that acetate can be used in place of cofactor AcCoA to carry out the cm^5^U modification reaction, and that blocking the molecular tunnel disrupts Elp3-mediated tRNA modification activity without significantly affecting tRNA binding or AcCoA binding and turnover. Together these data suggest a new model for Elp3-mediated tRNA modification in which the acetyl group from AcCoA is delivered to the rSAM active site via transport of free acetate molecules through a conserved, 20+ Å-long molecular tunnel connecting both domains of Elp3. We propose that conserved residues in the rSAM active site that line the molecular tunnel may then noncovalently coordinate and position acetate for sequential reaction with 5′-dA· and tRNA U34 to catalyze cm^5^U formation on tRNA. This tRNA modification mechanism suggests Elp3 might be therapeutically targeted by inhibitors mimicking acetate intermediates, reveals the first example of acetate transport between enzyme active sites, and shows how molecular tunnels may be formed by protein-nucleic acid interactions in rSAM enzymes.

## Results

### Elp3-tRNA complexes form a conserved molecular tunnel connecting the rSAM active site and AcCoA binding site

**Fig. 1:**
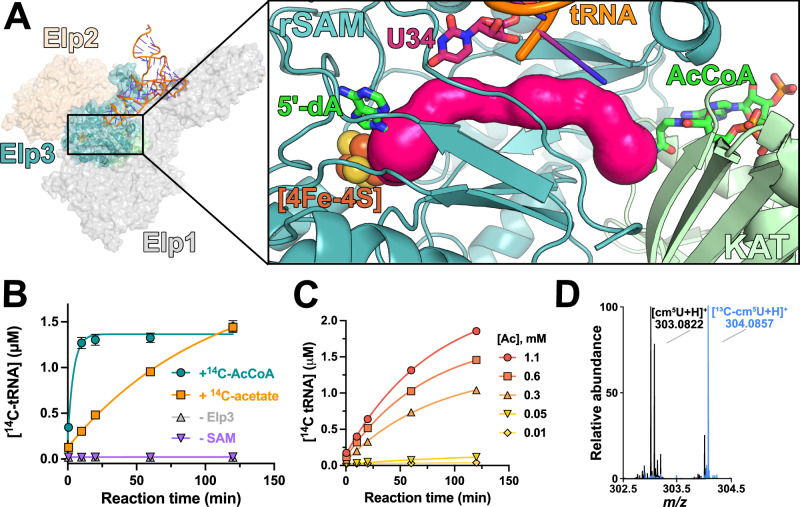
Elp3 uses a molecular tunnel to transport acetate to the rSAM active site for tRNA modification. **A** CAVER analysis of the yeast Elp1-Elp2-Elp3 complex bound to tRNA (PDB 8ASW) reveals an enclosed molecular tunnel connecting the Elp3 rSAM active site and the KAT domain AcCoA binding site (inset; CAVER-calculated tunnel shown in pink). The Elp3 rSAM KAT domains are colored teal and light green, respectively; 5′-dA and AcCoA analog desulfo-CoA (aligned from PDB 6IA6) are shown as green sticks. tRNA and U34 are shown in orange and pink, respectively. **B** Upon incubation with substrate tRNA Arg^UCU^ and ^14^C-labeled acetate (orange squares), *Min* Elp3 carboxymethylates tRNA in similar quantities as the enzyme does with ^14^C-labeled AcCoA (teal circles) after 2 hours, while negative controls without Elp3 (gray triangles) or SAM (purple triangles) produce no radiolabeled tRNA product. Data were fit to a single-phase exponential equation and are shown as mean values ± SD (*n* = 3). **C** Incubation of *Min* Elp3 with substrate tRNA Arg^UCU^ and increasing concentrations of ^14^C-acetate results in increasing amounts of ^14^C-labeled tRNA product formation. Data were fit to a single-phase exponential equation. **D** LC-MS analysis confirms that Elp3 forms ^12^C-containing (i.e., non-isotopically labeled) cm^5^U from ^12^C acetate in vitro (black spectrum), while an activity assay with ^13^C-labeled acetate forms ^13^C-labeled cm^5^U with an observed +1 Da mass shift in the cm^5^U nucleoside product (blue spectrum). Source data are provided as a Source Data file.

Elp3 uses cofactors SAM and AcCoA to catalyze formation of cm^5^U by radical-mediated incorporation of the acetyl group from AcCoA into tRNA. In their 2014 study, Selvadurai et al. carried out isotope incorporation experiments using d_3_-acetyl-CoA that showed the 5′-dA· generated in the rSAM site directly abstracts an H atom (or D atom in the case of d_3_-AcCoA) from AcCoA’s acetyl group^31^. However, every structure of Elp3 alone or in the context of the Elongator complex determined to date shows a 20+ Å distance between the rSAM active site where 5′-dA· is generated and the KAT domain where AcCoA is bound^30,32–35^, raising the critical question of how the acetyl group from AcCoA comes close enough to react with 5′-dA·. Using a previously determined cryo-EM structure of tRNA-bound Elp3 from the yeast Elongator complex (PDB 8ASW)^30^, we performed a structural analysis using CAVER 3.0, which maps and analyzes tunnels and channels in protein structures^48^. Our analysis revealed that the Elp3-tRNA complex forms an enclosed molecular tunnel reaching from the AcCoA binding site on the KAT domain directly to the [4Fe-4S] cluster and SAM binding site in the rSAM domain (Fig. 1A). We find that similar enclosed, molecular tunnels connecting the rSAM and KAT sites are found in archaeal and mammalian Elp3-tRNA complex models (Supplementary Fig. 3), suggesting that the molecular tunnel feature is widely conserved across all domains of life. Formed mostly by the rSAM domain’s partial TIM barrel motif, the tunnel has additional surfaces provided by both the KAT domain and the substrate tRNA. The tunnel-lining residues are highly conserved, composed almost entirely of either identical or chemically similar residues in diverse Elp3 sequences (Supplementary Fig. 4). In the yeast Elp3-tRNA structure, the diameter of the tunnel ranges from 3.4–6.4 Å, suggesting the possibility that small molecules could diffuse or be transported along its length.

### Elp3 uses acetate as a key intermediate to form cm^5^U34

Based on our structural analysis with CAVER and discovery of the conserved molecular tunnel, we hypothesized that Elp3 might resolve the large distance between the [4Fe-4S]/SAM and AcCoA binding sites by generating free acetate (or acetic acid, depending on local pH) from AcCoA at the KAT domain, transporting the acetate molecule through the tunnel, and delivering it for reaction with 5′-dA· and tRNA in the rSAM active site. To experimentally test this and other mechanistic hypotheses, we first overexpressed and anaerobically purified archaeal *Methanocaldococcus infernus* (*Min*) Elp3 and then screened [4Fe-4S] cluster reconstitution and reduction conditions in order to obtain in vitro tRNA-modifying activity. We verified the presence of [4Fe-4S] cluster in reconstituted Elp3 by UV-vis absorption at 420 nm (Supplementary Fig. 5) and then carried out radiolabel-based kinetic experiments using ^14^C-AcCoA to monitor Elp3-mediated tRNA modification over time. Under optimized anaerobic reaction conditions, we obtained robust and reproducible Elp3- and SAM-dependent tRNA modification activity (Fig. 1B) and validated formation of cm^5^U on tRNA by LC-MS and MS/MS (Supplementary Fig. 6).

With catalytically active recombinant Elp3 in hand, we next tested if acetate was able to replace AcCoA in our ^14^C-based tRNA modification assays. Using ^14^C-labeled acetate in place of ^14^C-AcCoA, we found that Elp3 could still carry out the tRNA modification reaction (Fig. 1B, orange). The somewhat slower reaction rate observed for acetate- versus AcCoA-based Elp3 activity may reflect the fact that reactions carried out with AcCoA, where acetate is released into the enclosed molecular tunnel, are likely able to achieve a dramatically higher local concentration of acetate in the rSAM active site, compared to reactions carried out with only exogenous acetate. Indeed, with a calculated Elp3 tunnel volume of 370 ± 30 Å^3^, one molecule of acetate released into the enclosed molecular tunnel would have an effective concentration of approximately 4.5 M, much higher than the 2 mM ^14^C-acetate used in these reactions. Consistent with this, we also found that the amount of ^14^C-tRNA product formed was dependent on the ^14^C-acetate concentration (Fig. 1C) and increased linearly with ^14^C-acetate concentrations up to 2 mM (Supplementary Fig. 7), suggesting that acetate is very weakly bound within the rSAM active site. We next confirmed that cm^5^U was indeed being formed on tRNA during these reactions using unlabeled acetate and LC-MS (Fig. 1D, black spectrum; Supplementary Fig. 8). Additionally, we carried out tRNA modification reactions using ^13^C-labeled acetate followed by LC-MS and observed a +1 Da mass unit shift in the resulting cm^5^U nucleoside product (Fig. 1D, blue spectrum; Supplementary Fig. 8), which unambiguously demonstrates that the added acetate is the reactive species that Elp3 uses to modify tRNA in these assays. Together, these biochemical data show that Elp3 can entirely bypass the need for cofactor AcCoA in its tRNA modification reaction and instead use molecular acetate to catalyze cm^5^U formation.

### Blocking the molecular tunnel inactivates Elp3

**Fig. 2:**
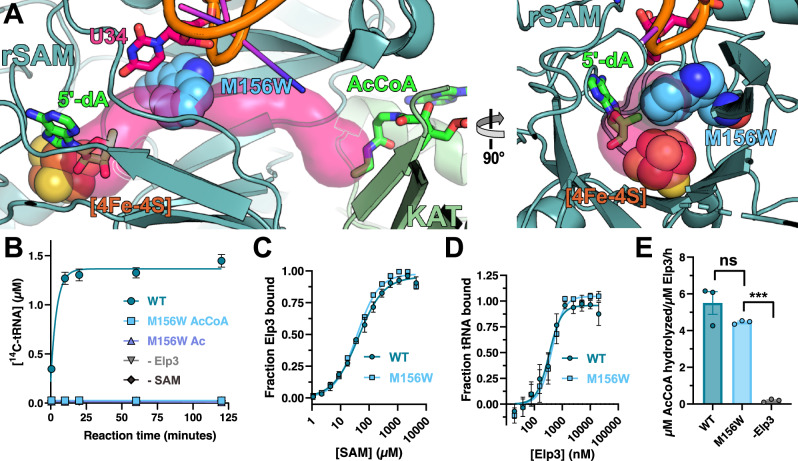
The Elp3 M156W mutation blocks the molecular tunnel and eliminates tRNA-modifying activity without disrupting SAM binding, tRNA binding, or AcCoA hydrolysis. **A** Mutagenesis modeling and analysis of Elp3 bound to tRNA (from PDB 8ASW) in PyMOL suggested that the M156W mutation (shown in blue spheres), located midway along the molecular tunnel, will entirely block Elp3’s tunnel and likely impede diffusion of acetate from the KAT AcCoA binding site to the rSAM active site. **B** Incubation of Elp3 M156W with substrate tRNA Arg^UCU^ and ^14^C-labeled AcCoA (light blue squares) or ^14^C-labeled acetate (purple triangles) in vitro results in no observable tRNA modification activity after 2 hours compared to WT *Min* Elp3 (teal circles); activity of the M156W mutant is comparable to -Elp3 (gray triangles) and -SAM (dark brown diamonds) negative controls. Data were fit to a single-phase exponential equation and are shown as mean values ± SD (*n* = 3). **C** Elp3 M156W (light blue squares) binds cofactor SAM with comparable affinity to WT Elp3 (teal circles). Elp3-SAM binding was measured by triplicate microscale thermophoresis (MST) assays, and fraction bound data were fit to a hill binding model and are shown as mean values ± SEM (*n *= 3). **D** Elp3 M156W (light blue squares) binds substrate tRNA Arg^UCU^ with comparable affinity to WT Elp3 (teal circles). Elp3-tRNA binding was measured by triplicate electrophoretic mobility shift assays (EMSAs; Supplementary Fig. 12), and fraction bound data were fit to a hill binding model and are shown as mean values ± SEM (*n* = 3). **E** Elp3 M156W (light blue) has similar AcCoA hydrolysis activity in the presence of substrate tRNA Arg^UCU^ compared to WT Elp3 (teal); both WT and M156W Elp3 show more AcCoA hydrolysis activity than a -Elp3 negative control (gray). AcCoA hydrolysis was measured in triplicate with a commercial fluorometric AcCoA/CoA quantification kit and statistical significance was calculated using a one-way ANOVA with Dunnett’s multiple comparisons test (*** *p *≤  0.001, ns *p* > 0.05); errors shown as mean values ± SEM (*n* = 3). Source data are provided as a Source Data file.

If Elp3 uses a tunnel to transport molecular acetate from the KAT domain to the rSAM domain, sterically occluding the tunnel to restrict acetate transit should lead to diminished tRNA modification activity. Using the yeast Elp3-tRNA structure, we modeled how potential point mutations that increase the size of tunnel-lining residues might constrict or block the tunnel; we specifically chose non-conserved residues for this analysis to minimize the likelihood that potential tunnel-blocking mutations would disrupt other functions of Elp3. We identified the M156W mutation as one that would be predicted to entirely block the molecular tunnel midway between the KAT and rSAM sites (Fig. 2A). Elp3 M156W was expressed, purified, and reconstituted using the same anaerobic conditions as for wild-type Elp3, resulting in purified recombinant enzyme that has a very similar size exclusion chromatography profile and UV-vis absorbance spectra as WT Elp3 (Supplementary Fig. 9). tRNA modification assays with either ^14^C-AcCoA or ^14^C-acetate reveal that the M156W variant completely eliminates Elp3-mediated tRNA modification activity (Fig. 2B). Critically, although M156W is located in relatively close proximity to the tRNA backbone in the Elp3-tRNA structure, this mutation does not significantly impact SAM binding to the rSAM active site (Fig. 2C), tRNA binding to Elp3 (Fig. 2D), or the binding and turnover of AcCoA (Fig. 2E), which is thought to be somehow coupled to formation of the Elp3-tRNA complex^31,33^. Similar results were obtained with an additional tunnel-blocking mutation, Elp3 I154W (Supplementary Fig. 9 and 10), and AlphaFold3 predictions suggest that neither of these mutations impact the overall protein fold (Supplementary Fig. 11). Together, these mutagenesis, biochemical, and biophysical experiments suggest that blocking the molecular tunnel inactivates Elp3 tRNA modification catalysis by preventing the passage of acetate from the KAT domain into the rSAM active site after tRNA binding and AcCoA hydrolysis. Furthermore, because tunnel-blocked Elp3 mutants are still inactive in reactions with added acetate, these data also suggest that acetate cannot effectively access the rSAM active site directly, but must pass through the tunnel and into the structured and tRNA-bound active site for efficient tRNA modification activity.

### Nucleophilic or hydrolytic reaction of acetate is not required for Elp3-mediated tRNA modification

**Fig. 3:**
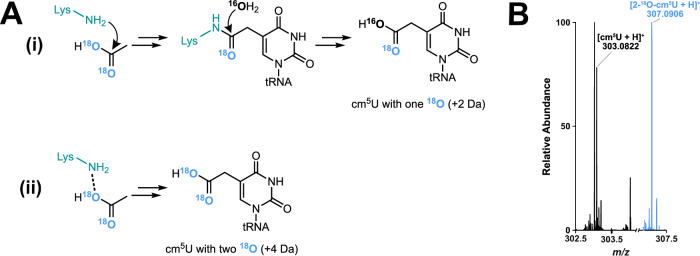
Elp3 forms doubly ^18^O-labeled cm^5^U tRNA from ^18^O_2_-acetate. **A** (**i**) If acetate undergoes nucleophilic attack during the Elp3-mediated reaction to form an acetylated residue, the reaction would displace one of the ^18^O isotope labels. Subsequent hydrolysis of the acetylated residue by bulk (^16^O, non-labeled) water would result in a cm^5^U product with one ^16^O and one ^18^O label in the carboxylate and a +2 Da mass unit shift above normal isotopic abundance cm^5^U. (**ii**) In contrast, noncovalent coordination of free acetate would result in both ^18^O labels being retained at the carboxylate and a +4 Da mass unit shift above normal isotopic abundance cm^5^U. **B** LC-MS analysis confirms that Elp3 forms doubly ^18^O-labeled cm^5^U from ^18^O_2_-acetate in vitro, with the resulting cm^5^U product (blue spectrum) showing a +4 Da mass shift compared to cm^5^U formed using normal isotopic abundance acetate (black spectrum). The normal abundance activity assay was performed with 5 µM Elp3, 4.4 µM tRNA, 25 µM SAM, and 10 mM normal isotopic abundance acetate and the ^18^O-acetate activity assay was performed with 25 µM Elp3, 4.4 µM tRNA Arg^UCU^, 50 µM SAM, and 10 mM ^18^O_2_-acetate.

Previously proposed mechanisms^30,31,35^ have suggested the possibility that acetylation of key Elp3 residues might be required to transport or help position the acetyl group in the rSAM active site for tRNA modification. To determine whether or not acetate forms an intermediate acetylated residue or undergoes hydrolysis during the course of the tRNA modification reaction, we performed an isotope incorporation experiment using doubly ^18^O-labeled acetate. If acetate must undergo nucleophilic addition and/or hydrolysis at any point during the reaction mechanism, this should result in the obligate loss of at least one of the two ^18^O isotope labels in the final cm^5^U product (Fig. 3A, i). In contrast, if Elp3 uses only noncovalent coordination of free acetate to carry out the modification reaction, this would result in both ^18^O labels being retained in the final cm^5^U product (Fig. 3A, ii). We carried out tRNA modification reactions using doubly ^18^O-labeled acetate and observed a peak corresponding to cm^5^U nucleoside product with a +4 Da mass unit shift, consistent with formation of cm^5^U that retains both ^18^O labels (Fig. 3B, Supplementary Fig. 13). While peaks corresponding to non-labeled and singly ^18^O-labeled cm^5^U product are also present (Supplementary Fig. 14), this is likely due to oxygen’s known tendency to undergo exchange at carboxylate positions in water^49,50^. The clear presence of doubly ^18^O-labeled cm^5^U product in Elp3 reactions carried out with ^18^O_2_-acetate strongly suggests that acetate is not required to undergo nucleophilic or hydrolytic reaction during the tRNA modification reaction cycle and is instead likely positioned in the rSAM active site through noncovalent interactions.

### Conserved Elp3 rSAM active site residues in the molecular tunnel may position acetate for radical-mediated reaction

**Fig. 4:**
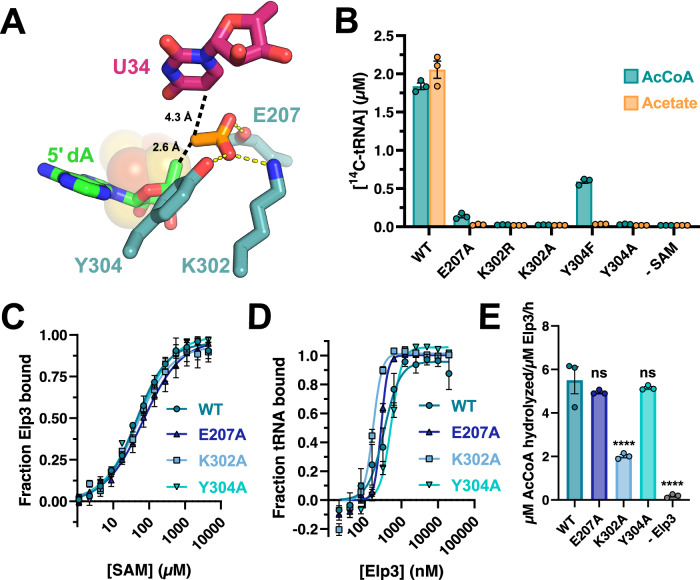
Molecular docking and activity assays reveal tunnel-lining active site residues critical for Elp3-mediated tRNA-modifying activity. **A** SeamDock model of acetate bound in Elp3’s rSAM active site using the yeast Elp3-tRNA structure (PDB 8ASW), with *Min* Elp3 numbering. 5′-dA is shown in green, U34 is pink, and the [4Fe-4S] cluster is shown as spheres. Yellow dotted lines denote hydrogen bond or ionic interactions between 2.9 and 3.2 Å; black dotted lines show the distances between the acetate methyl carbon and either the 5′ carbon of 5′-dA, or the C5 carbon of tRNA U34. **B** Comparison of WT Elp3 activity with ^14^C-AcCoA or ^14^C-acetate to E207, K302, and Y304 mutants reveals that E207A, K302R, K302A, and Y304A display no or nearly no activity with AcCoA or acetate, while Y304F retains partial activity with AcCoA. Data are shown as mean values ± SEM (*n* = 3). **C** Elp3 E207A (dark blue triangles), K302A (light blue squares), and Y304A (cyan triangles) mutants bind cofactor SAM with comparable affinity to WT Elp3 (teal circles). SAM binding was measured in triplicate using MST, with data fit to a hill binding model and shown as mean values ± SEM (*n* = 3). **D** Elp3 variants bind substrate tRNA Arg^UCU^ with similar affinities to WT Elp3; the same color and labeling scheme for WT and Elp3 mutants are used as in C. Elp3-tRNA binding was measured by triplicate electrophoretic mobility shift assays (EMSAs; Supplementary Fig. 18). Data were fit to a hill binding model and are shown as mean values ± SEM (*n* = 3). **E** E207A (dark blue) and Y304A (cyan) Elp3 mutants exhibit similar AcCoA hydrolysis activity in the presence of substrate tRNA Arg^UCU^ compared to WT Elp3 (teal), whereas K302A (light blue) has moderately reduced hydrolysis activity; all mutants and WT Elp3 show more AcCoA hydrolysis activity than a -Elp3 negative control (gray). Statistical significance was calculated using a one-way ANOVA with Dunnett’s multiple comparisons test (**** *p* < 0.0001, ns *p* > 0.05); data are shown as mean values ± SEM (*n* = 3). Source data are provided as a Source Data file.

Based on the ^18^O labeling experiments above, we presumed that a number of conserved rSAM active site residues of Elp3 would be used to noncovalently coordinate and position acetate for H atom abstraction by 5′-dA· and subsequent addition to tRNA U34. Although we have not yet succeeded in obtaining acetate-bound Elp3 structures, we used the molecular docking programs CaverDock^51^ and SeamDock^52^ to visualize and generate hypotheses about how acetate might be bound at different locations along the Elp3 molecular tunnel and within the rSAM active site (Supplementary Fig. 15). From these computational tools, we obtained predicted poses of acetate that interact with several strictly conserved, tunnel-lining Elp3 active site residues (*Min* residues E207, K302, Y304; see Supplementary Table 1 for eukaryotic residue numbering), and at the same time position the acetate methyl group between the 5′ carbon atom of 5′-dA and the C5 position of U34 tRNA, which would allow direct H atom abstraction and radical transfer from 5′-dA· to the acetate methyl group and then to tRNA U34 (Fig. 4A). While these are only rough models based on simple docking algorithms, the predicted acetate poses suggest that acetate can reasonably be accommodated at many positions along the molecular tunnel and may be oriented within the active site by noncovalent interactions with conserved tunnel-lining residues to facilitate sequential reaction with 5′-dA· and tRNA U34.

To provide experimental support for these predictions, we mutated the conserved, tunnel-lining rSAM active site residues E207, K302, and Y304 (Supplementary Fig. 16), which make hydrogen bond contacts with acetate in the docking models, and measured their relative tRNA modifying, SAM binding, tRNA binding, and AcCoA turnover capacities (Fig. 4B–E). In reactions with ^14^C-AcCoA (Fig. 4B, teal bars), all mutations to E207 (E207A) or K302 (K302R, K302A) resulted in nearly complete loss of tRNA modifying activity relative to WT Elp3. For Y304, while the Ala mutant Y304A resulted in complete loss of activity, the Phe mutant Y304F retained about 30% activity relative to WT, showing that while the Y304 hydroxyl group is important for Elp3 reactivity with AcCoA, it is not essential. We next similarly tested reactions of Elp3 with ^14^C-acetate (Fig. 4B, orange bars) and found that for acetate-based tRNA modification reactivity, all of the tested Elp3 mutants were inactive. AlphaFold3 models predict that these mutations do not disrupt the overall protein fold (Supplementary Fig. 17). These mutational data show that both AcCoA- and acetate-based Elp3 reactivity are critically sensitive to the same rSAM active site tunnel mutations, consistent with our proposed mechanism in which molecular acetate is a key reaction intermediate in the Elp3-catalyzed tRNA modification reaction. The lower activity observed for acetate- versus AcCoA-based reactivity with Elp3 mutant Y304F may reflect the lower effective local concentration of acetate achieved in reactions with exogenously added acetate versus reactions with AcCoA (as discussed above), making these reaction conditions more sensitive to mutations that likely disrupt acetate binding to the rSAM site.

We next carried out microscale thermophoresis (MST)-based assays to assess cofactor SAM binding (Fig. 4C) and electrophoretic mobility shift assays (EMSAs) to assess substrate tRNA binding (Fig. 4D) to the Elp3 mutants. We found that none of the tested mutations altered SAM binding to the rSAM active site, and the only mutation that moderately weakened tRNA binding was Y304A, which increased *K*_D_ by approximately 1.5-fold compared to WT. Finally, we also tested the impact of these Elp3 mutations on AcCoA turnover (Fig. 4E) and found that E207A and Y304A mutations had no effect compared to WT, while K302A moderately decreased AcCoA hydrolysis by approximately 3-fold. Together with the ^14^C-based activity assays above, and consistent with past studies of Elp3 and Elongator, these data show that active site residues K302 and Y304 play critical roles in tRNA modification reactivity and moderately impact a combination of tRNA binding, tRNA positioning, and/or AcCoA turnover. These residues may play multifunctional roles in the Elp3 active site, also helping to bind or position acetate for radical reaction, although we have thus far been unable to develop a robust acetate binding assay that might help to decouple these different functions. The functional properties of conserved rSAM active site residue E207 have not previously been investigated, but our data show that while this residue is essential for both AcCoA- and acetate-based tRNA modification activity, its mutation has no deleterious impacts on SAM binding, tRNA binding, or AcCoA hydrolysis. In the framework of our proposed model of Elp3 catalysis, this suggests E207 may play an important role in the positioning of intermediate acetate within the tunnel and rSAM active site. However, high-resolution structures of Elp3 with acetate will likely be required to unambiguously determine the atomic-level roles of tunnel-lining active site residues during key reaction steps.

## Discussion

**Fig. 5:**
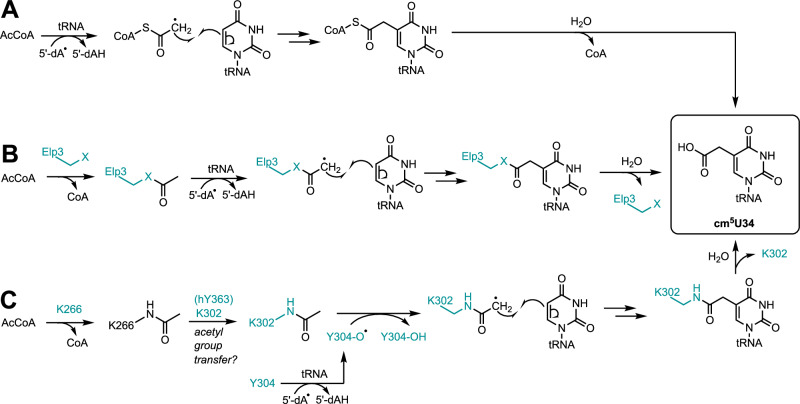
Previously proposed Elp3 tRNA modification mechanisms. **A**, **B** are mechanisms originally proposed by Selvadurai et al. in 2014^31^, characterized by either reaction of AcCoA directly with 5′-dA· (**A**), or initial acetylation of Elp3 by AcCoA followed by reaction of the acetylated Elp3 residue with 5′-dA· (**B**). **C** a variation on (**B**) more recently proposed by Abassi et al. in 2024^35^, in which AcCoA is used to acetylate a conserved lysine, this covalent acetyl group is then transferred from residue to residue along Elp3, to ultimately react with a tyrosyl radical in the rSAM active site.

Elp3 and Elongator install the key cm^5^U34 modification on the anticodon loop of tRNA, a critical intermediate for the subsequent formation of a whole family of cm^5^U-derived tRNA modifications (Supplementary Fig. 1) that have diverse impacts on cellular translational efficiency and fidelity across all domains of life. Over the past decade, multiple mechanisms have been proposed to explain how this functionally important tRNA modification is installed by the rSAM and KAT domains of Elp3. In 2014, Selvadurai et al. proposed two possible, general mechanisms for Elp3-mediated tRNA modification based on isotope incorporation experiments (Fig. 5): (A) 5′-dA· generated at the rSAM active site abstracts a hydrogen atom directly from AcCoA to form an AcCoA-based radical that subsequently reacts with tRNA U34 resulting in an AcCoA-tRNA covalent intermediate that is hydrolyzed to ultimately produce cm^5^U (Fig. 5A). Alternatively, (B) AcCoA first acetylates an unspecified Elp3 residue, 5′-dA· abstracts a hydrogen atom from the acetylated Elp3 residue to generate an acetyl-based radical, which reacts with tRNA U34 to produce a covalent Elp3-tRNA adduct, and finally hydrolysis of the acetylated Elp3 residue produces cm^5^U (Fig. 5B). Since 2019, multiple structures of Elp3 and Elongator subunits in complex with tRNA have been determined^30,34,35^, and based on this structural data Abbassi et al. have more recently proposed a variation on mechanism (B) above, in which (C): AcCoA acetylates a conserved Elp3 lysine residue, the acetyl group is somehow passed from residue to residue along Elp3, to ultimately produce an acetylated lysine (*Min* K302, human K316; Supplementary Table 1) in the rSAM active site of Elp3. In this mechanism, 5′-dA· is proposed to abstract a hydrogen atom from an active site tyrosine (*Min* Y304, human Y318; Supplementary Table 1), the resulting tyrosyl radical reacts with the acetylated active site lysine, and the acetyl lysine radical then reacts with tRNA U34 (Fig. 5C)^35^. However, in large part due to challenges in obtaining active recombinant Elp3, none of these mechanisms have been subjected to enzymological investigations in vitro that might more clearly define the biochemical mechanism of Elp3- and Elongator-mediated tRNA modification.

**Fig. 6:**
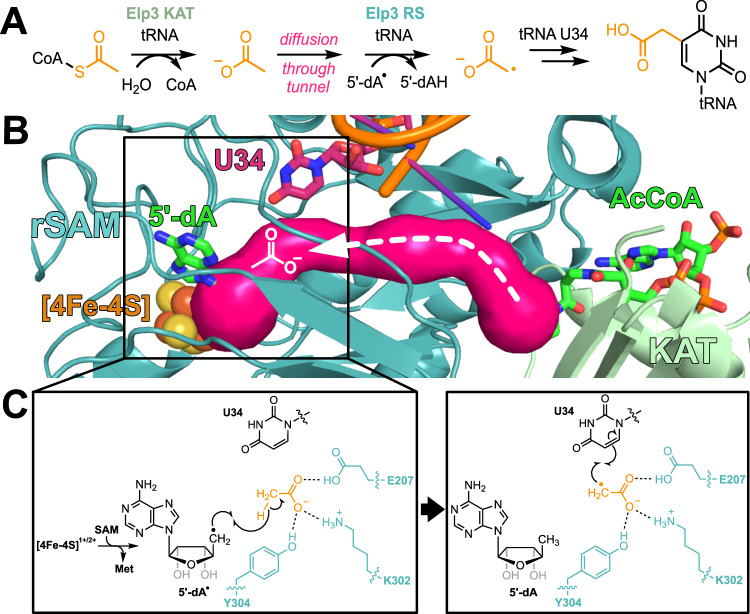
Summary of Elp3 tRNA modification reaction mechanism involving transport of acetate through the Elp3 molecular tunnel. **A** Overview of proposed Elp3 tRNA modification reaction. Elp3 hydrolyzes AcCoA in the KAT site to produce acetate (or acetic acid, depending on local pH), which then diffuses through Elp3’s molecular tunnel to the rSAM active site. Once acetate is in the rSAM site, 5′-dA∙ abstracts a hydrogen atom from acetate to produce an acetate radical, which adds to the C5 position of U34 on substrate tRNA to form cm^5^U. **B** Structural overview of proposed Elp3 tRNA-modifying reaction using the yeast structure of Elp3 bound to tRNA (PDB 8ASW). The Elp3 rSAM domain is colored teal, the Elp3 KAT domain is colored light green, and the calculated tunnel is shown in pink; 5′-dA and AcCoA analog desulfo-CoA (aligned from PDB 6IA6) are shown in green sticks. tRNA and substrate tRNA base U34 are shown in orange and pink, respectively. **C** Schematic of acetate’s reaction with 5′-dA· and U34 in the rSAM active site. Left: key conserved rSAM active site residues (teal) noncovalently position acetate (orange) for hydrogen atom abstraction by 5′-dA· . Right: next, the coordinated acetate radical adds to the C5 position of substrate tRNA U34 to ultimately form cm^5^U-modified tRNA. All Elp3 residues are shown with *Min* numbering; see Supplementary Table 1 for eukaryotic residue numbering.

Here we have chemically reconstituted the [4Fe-4S] cluster and obtained robust in vitro tRNA modification activity for a model, recombinant archaeal Elp3. Our structural analysis of Elp3-tRNA complexes revealed a conserved molecular tunnel connecting the rSAM and KAT active sites on Elp3 (Fig. 1A, Supplementary Fig. 3), suggesting a new possible mechanism for Elp3-mediated tRNA modification where the molecular tunnel might be used to transport free acetate (or acetic acid) molecules between the distant Elp3 active sites. In support of this hypothesis, our in vitro kinetic assays with reconstituted Elp3 showed that acetate can be used in place of AcCoA to carry out cm^5^U formation on tRNA, and that the CoA portion of AcCoA is not needed to carry out the tRNA modification reaction at sufficient acetate concentrations (Fig. 1B–D). Sterically blocking the molecular tunnel by mutagenesis disrupts Elp3-mediated tRNA modification activity without significantly affecting SAM binding, tRNA binding, or AcCoA binding and turnover (Fig. 2, Supplementary Fig. 10). Similar experiments mutating tunnel-lining residues to either block or open tunnels have been used to verify and elucidate the functions of molecular tunnels that transport small molecules or gases in a variety of other enzymes^53–59^. Based on our structural analysis and biochemical data, we therefore propose a new model (Fig. 6) for tRNA modification by Elp3 whereby the acetyl group from AcCoA is delivered to the rSAM active site via transport of free acetate through a conserved, 20+ Å-long molecular tunnel connecting both domains of Elp3.

Approximately a dozen known enzymes use a molecular tunnel to connect two distant active sites within the enzyme^58,60–62^, most commonly for the transport of ammonia^61^, though examples of indole^63^, carbon monoxide^64^, dehydroglycine^62,65^, and acetaldehyde^66^ transport are known. Multisite tunnel enzymes appear to have evolved their tunnels independently, but some share convergent similarities such as a similar makeup of conserved tunnel-lining residues and similar tunnel lengths of ~20-30 Å^61^. To our knowledge, Elp3 and Elongator would be the only known enzyme or enzymatic complex to use a tunnel for the transport of molecular acetate between multiple active sites. The Elp3 tunnel is composed largely from its partial TIM barrel motif, a feature with similarity to tunnel-containing enzymes imidazole glycerolphosphate synthase (IGPS)^67,68^ and fellow rSAM enzyme HydG^62,65^, both of which use full TIM barrels to transport intermediates, whereas Elp3’s partial TIM barrel is capped by tRNA to complete the tunnel. That multiple enzymes utilize a TIM barrel or partial TIM barrel in their molecular tunnels is hardly surprising, as the tubular interior of the motif’s architecture readily lends itself to small molecule or ion transport. Across the huge radical SAM enzyme superfamily however, Elp3 would be only the third identified rSAM enzyme that uses a molecular tunnel to transport substrates or intermediates; the only other known examples being HydG, mentioned above, and the closely related enzyme ThiH, both of which transport dehydroglycine as a reaction intermediate using enclosed tunnels^62^. Elp3 is the first example where the tunnel is formed in part by RNA, and suggests that other rSAM enzymes may also form molecular tunnels in complex with additional protein cofactors or nucleic acid. The rSAM superfamily is incredibly diverse, with over 700,000 unique sequences identified^69^, less than 4000 of which have been functionally characterized^70^ and less than 100 of which have been structurally characterized^71,72^. We therefore anticipate that other tunnel-using rSAM enzymes likely exist and have yet to be discovered due to their large, but largely unexplored, diversity and conserved architectures featuring a full or partial TIM barrel motif^72,73^.

Molecular docking simulations using CaverDock and SeamDock to model possible acetate positions within the Elp3 molecular tunnel suggested acetate poses in the Elp3 rSAM active site where the acetate methyl group is positioned directly between the 5′ carbon of 5′-dA and the C5 carbon of tRNA U34, at distances reasonable for radical-mediated reactivity between each of these reaction components (Fig. 4A, Supplementary Fig. 15). This docking pose also places acetate within hydrogen bonding distance to key conserved, tunnel-lining active site residues E207, K302, and Y304. Mutating these rSAM active site residues results in inactivation of Elp3 for AcCoA- and acetate-based reactions in all cases except for the Y304F mutation with AcCoA, which retains partial activity (Fig. 4B). K302A and Y304A mutations showed moderate defects in AcCoA hydrolysis (three-fold) or tRNA binding (1.5-fold), respectively, whereas E207A had no significant impacts on SAM binding, tRNA binding, or AcCoA hydrolysis (Fig. 4C–E), suggesting a possible key role for E207 in acetate positioning within the tunnel. These data are broadly consistent with previous mutagenesis experiments in yeast-based assays, showing K302 (*Sc* K325) and Y304 (*Sc* Y327) are important for Elongator function^30,34,35^. Our experiments additionally reveal the importance of E207, which has not previously been characterized, and show that AcCoA- and acetate-based reactivity are similarly dependent on the same subset of rSAM active site residues. Our observation that the Y304F mutation retains partial activity with AcCoA is also consistent with previous isotope incorporation experiments showing direct reaction of the AcCoA acetyl group with 5′-dA·^31^, in contrast to recent proposals suggesting intermediate radical transfer through Y304 (human Y318), which would strictly require the tyrosyl hydroxyl group^30,35^. Finally, our isotope incorporation experiments showing that reaction of doubly ^18^O-labeled acetate results in formation of doubly ^18^O-labeled cm^5^U suggest that acetate is not required to undergo nucleophilic or hydrolytic reaction during Elp3’s catalytic cycle, further supporting the idea that Elp3 transports and uses free acetate rather than an acetylated residue intermediate.

Because the Elp3 molecular tunnel, as well as the key tunnel-lining and active site residues identified here as critical for both AcCoA- and acetate-based tRNA modification, are conserved across all domains of life, we propose that the acetate transport mechanism described here for archaeal Elp3 (Fig. 6) is also likely operative in the eukaryotic Elongator complex. Notably, several cancer-related human Elp3 somatic missense variants identified in tumor sequencing datasets (TCGA)^74^ or reported previously^35^ – Y127C, M170I, R242K, and E244D/Q – map to residues lining the Elp3 molecular tunnel (Supplementary Fig. 19). The R242K mutation was previously shown to decrease AcCoA hydrolysis by ~three-fold in human Elongator, and we show in this paper that *Min* M156W (corresponding to human M170) mutations cripple Elp3 activity, suggesting the tumor-related mutation M170I could have impacts on Elp3 activity as well. Future characterization of these cancer-related missense variants will be needed to understand how they may impact Elongator activity and to assess their potential functional significance in human cancers.

Together, our biochemical data and structural analyses show how Elp3, and by extension Elongator, use a conserved molecular tunnel to transport acetate from the Elp3 KAT domain to the rSAM active site, where acetate is likely positioned through key noncovalent interactions to undergo reaction with 5′-dA· and then tRNA U34 to catalyze formation of the cm^5^U modification on tRNA. Our proposed mechanism for Elp3- and Elongator-mediated tRNA modification answers long-standing questions about how the enzyme bridges the large distance between the AcCoA binding site on the KAT domain and the [4Fe-4S]-containing active site in the rSAM domain. This provides important groundwork toward understanding how tRNA and other Elp proteins in the Elongator complex may help regulate tRNA modification chemistry and how the active site and/or tunnel of Elp3 could be targeted with small molecules mimicking acetate. More broadly, this work elucidates a novel tRNA modification and acetate transport mechanism and suggests that the rSAM enzyme superfamily may use diverse, yet-to-be-discovered molecular tunnels to carry out intermediate transport and reactivity.

## Methods

### CAVER, CaverDock, and SeamDock analysis

Analysis of the tRNA-bound yeast Elp3 structure (from PDB 8ASW)^30^ was performed with the CAVER 3.0.3 PyMOL plugin^48^. CAVER settings were as follows: probe radius 1.4 Å, starting coordinates *x* = 186.918, *y* = 158.706, *z* = 197.689, and input atoms included all 20 amino acids and all 4 RNA bases, but not 5′-dA or the [4Fe-4S] cluster. The model of yeast Elp3 with tRNA bound derived from PDB 8ASW and the CAVER tunnel generated using the above were used as CaverDock inputs. A model of acetate was obtained from the PDB^71^. The CAVER tunnel was discretized into discs with 0.3 Å between individual discs and the direction of tunnel analysis was reversed to model binding the acetate ligand from the tunnel’s entrance at the KAT site into the interior of Elp3. SeamDock analysis was performed on the tRNA-bound yeast Elp3 structure and an acetate model obtained from the PDB. The box center coordinates were set to *x* = 7, *y* = −6, z = 3 and all other settings were set to defaults.

### In vitro transcription (IVT) of Elp3 tRNA substrate

The *Methanocaldococcus infernus* (*Min*) tRNA^Arg^_UCU_ sequence (5′-GGACCCGUAGCCUAGCCUGGAUAGGGCACCGGCCUUCUAAGCCGGGGGUCGGGGGUUCAAAUCCCCCCGGGUCCGCCA-3′) was obtained from sequences deposited in the Genomic tRNA Database^75^. The corresponding tRNA^Arg^_UCU_ DNA template was PCR-amplified from a commercially obtained DNA oligonucleotide reverse complement sequence containing a 5′ T7 promoter sequence (Integrated DNA Technologies; template DNA sequence = 5′-TGGCGGACCCGGGGGGATTTGAACCCCCGACCCCCGGCTTAGAAGGCCGGTGCCCTATCCAGGCTAGGCTACGGGTCCTATAGTGAGTCGTATTA-3′). The tRNA was in vitro transcribed using T7 polymerase in 500 µL reactions: 0.25 µM DNA template, 2.5 mM rNTPs, 0.05% Triton X-100 (v/v), 5 µL of 10 mg/mL T7 polymerase, and 1 Unit of thermostable inorganic pyrophosphatase (New England Biolabs) in T7 buffer (40 mM Tris HCl, pH 7.5, 50 mM MgCl_2_, 2 mM spermidine, 5 mM dithiothreitol (DTT)). Reactions were incubated at 37 °C for 3 h, then the DNA template was digested with TURBO DNase (Thermo Fisher Scientific) for 1 h at 37 °C. Following this, tRNA was purified using denaturing urea PAGE gels, phenol:chloroform extracted, and precipitated with 80% ethanol at −20 °C. Isolated tRNA was redissolved in water, annealed for 2 min at 80 °C and 2 min at 60 °C, then MgCl_2_ was added to a final concentration of 10 mM and the tRNA was aliquoted, flash frozen with liquid nitrogen, and stored at −70 °C until ready for use.

### Cloning and overexpression of recombinant Min Elp3

The *Min* Elp3 gene (UniProt ID D5VRB9) was ordered as an *E. coli* codon-optimized DNA sequence (Integrated DNA Technologies) and cloned into a pET28a-tev vector containing an N-terminal 6xHis affinity tag and tobacco etch virus protease (TEV) cleavage site using Gibson assembly (New England Biolabs HiFi DNA Assembly Kit). The final expression vector sequence was confirmed by whole plasmid DNA sequencing (Plasmidsaurus). Elp3 point mutations were generated using site-directed mutagenesis by whole-plasmid polymerase chain reaction (PCR) and confirmed by DNA sequencing (Plasmidsaurus).

*Min* Elp3 plasmids and pDB1282 plasmid containing the *isc* operon were simultaneously transformed into *E. coli* BL21(DE3) cells. Bacterial growth was carried out in LB media at 37 °C with shaking. At an optical density of 600 nm (OD_600_) of 0.3–0.4, the *isc* operon was induced with a 20% (w/v) solution of L-(+)-arabinose for a final concentration of 0.2% (w/v). Solutions of 200 mM iron(III) chloride and 200 mM cysteine were added to a final concentration of 0.2 mM each. At an OD_600_ of 0.6-0.8, Elp3 expression was induced by adding 1 M IPTG to a final concentration of 1 mM. Induction occurred at 18 °C overnight with shaking. Cells were harvested at 6000 x *g* for 30 min at 4 °C, flash frozen with liquid nitrogen, and stored at −70 °C until ready for use.

### Purification of Min Elp3

Purification of *Min* Elp3 constructs was mostly carried out in an anaerobic chamber (MBraun) at an oxygen concentration of < 1 ppm and temperature of 16 °C. Buffers used during purification were as follows: lysis/wash buffer (50 mM HEPES, pH 7.5, 300 mM KCl, 2 mM imidazole, 10% glycerol (v/v)), wash 2 buffer (50 mM HEPES, pH 7.5, 1.0 M KCl, 10 mM imidazole, 10% glycerol (v/v)), wash 3 buffer (50 mM HEPES, pH 7.5, 300 mM KCl, 10 mM imidazole, 10% glycerol (v/v)), elution buffer (50 mM HEPES, pH 7.5, 300 mM KCl, 250 mM imidazole, 10% glycerol (v/v)), size exclusion chromatography (SEC) buffer (50 mM HEPES, pH 7.5, 300 mM KCl). All buffers were thoroughly deoxygenated by bubbling with argon for 1 h before being introduced into the anaerobic chamber and then stirred vigorously overnight to equilibrate. 10 mM 2-mercaptoethanol (BME) was added to buffers before use, with the exception of SEC buffer, which had 5 mM DTT added. Cell pellets were thawed inside the anaerobic chamber and resuspended in 35 mL of lysis/wash buffer, then lysed by sonication on ice using an airtight sonicator adaptor outside of the anaerobic chamber (Branson Sonifier 450, 4 × 2 min cycles at setting 6, 50% duty cycle). Following sonication, lysate was brought back into the anaerobic chamber and transferred to centrifuge tubes which were sealed and centrifuged outside of the anaerobic chamber for 45 min at 14,500 x *g* and 4 °C. Sealed centrifuge tubes were brought back into the anaerobic chamber and lysate was mixed for 1 h with Ni-NTA resin that had been deoxygenated by bubbling with argon gas and equilibrated with lysis/wash buffer. The mixture was transferred to a gravity column and the flow-through was collected. The resin was then washed with 50 mL of wash 2 buffer and 50 mL of wash 3 buffer, which were collected, before protein was eluted with 15 mL of elution buffer in 1 mL fractions. Following identification of Elp3-containing fractions by gel electrophoresis (10% polyacrylamide gel), fractions were pooled and concentrated to ~2 mL using Amicon Ultra 0.5 mL spin columns (50 kDa cutoff) and subjected to size exclusion chromatography on an AKTA Pure 25 M with a 16/60 S-200 pg column, pre-equilibrated with 3 column volumes of deoxygenated sizing buffer. SEC fractions were collected inside the anaerobic chamber.

### Chemical reconstitution of Min Elp3

Following identification of desired SEC fractions by gel electrophoresis (10% polyacrylamide gel), chosen fractions were concentrated and exchanged into reconstitution buffer (100 mM HEPES, pH 7.5, 500 mM KCl, 10% glycerol) using Amicon Ultra 0.5 mL spin columns (50 kDa cutoff) inside the anaerobic chamber. The purified Elp3 was then diluted to 75 µM in reconstitution buffer, with DTT added to a final concentration of 5 mM, and incubated for 1 h. Iron(III) chloride was gradually added to a 10-fold molar excess relative to Elp3 (6 aliquots, with 5 min between aliquot additions) and after 10 min of further incubation, sulfide (Na_2_S) was gradually added to a 10-fold molar excess (6 aliquots, with 15 min between aliquot additions). The reconstitution was allowed to proceed overnight. All reconstitution steps were carried out at 4 °C in the anaerobic chamber. Following reconstitution, Elp3 was centrifuged to remove any precipitate (25 min at 14,500 rpm, Eppendorf MiniSpin plus), buffer exchanged into reaction buffer (50 mM HEPES, pH 7.5, 150 mM KCl, 300 mM NaCl, 5 mM MgCl2, 1% glycerol), concentrated, glycerol was added to a final concentration of 20%, and flash frozen with liquid nitrogen. Elp3 aliquots were stored at -70 °C for further use. UV-Vis spectra were collected to verify the presence of the characteristic [4Fe-4S] absorption peak at 420 nm (Thermo Scientific NanoDrop 2000c).

### In vitro Elp3 activity assays

Substrate tRNA aliquots were brought into the anaerobic chamber and deoxygenated by equilibrating with the anaerobic atmosphere at 4 °C for 30 min. A 2X concentration Elp3 solution was prepared at 10 µM with 55 µM AcCoA, 50 µM SAM, 1 mM sodium dithionite, and 2X reaction buffer and incubated for 10 min at 16 °C. A 2X concentration tRNA solution was prepared by diluting tRNA aliquots to 8.8 µM with water. Reactions were initiated by mixing the 2X Elp3 and 2X tRNA solutions and incubated at 37 °C. Final reaction concentrations were 5 µM Elp3, 27.5 µM AcCoA, 25 µM SAM, 0.5 mM dithionite as a reductant, and 4.4 µM tRNA. Activity assays with acetate were carried out similarly, where the relevant concentration of acetate was used in place of AcCoA; 2 mM if not otherwise indicated. Elp3 active site variant activity was measured after a 4-hour incubation, whereas all the other activity assays were incubated for 2 h. 10 µL timepoint aliquots were quenched with 1 µL of 10 mM ethylenediaminetetraacetic acid (EDTA) stock solution. For each quenched timepoint, tRNA was then isolated from the other reaction components (Zymo RNA Clean and Concentrator kit) and eluted in 15 µL water. For radiolabel-based assays, 12 µL eluted tRNA was added to 10 mL of scintillation fluid (Ultima Gold) in a 20 mL glass scintillation vial and counted on a liquid scintillation counter (PerkinElmer Quantulus GCT 6220). All reported activity assays were carried out in triplicate, measured from separate samples. For mass spectrometry-based assays, eluted tRNA was digested into individual nucleosides (NEB Nucleoside Digestion Mix) and analyzed by LC-MS or LC-MS/MS. For the ^12^C- and ^13^C-acetate LC-MS/MS experiment, 10 mM acetate was used for the activity assay. For the ^18^O isotope incorporation experiment, the activity assay was performed with 25 µM Elp3, 10 mM ^18^O_2_- or normal abundance acetate, 50 µM SAM, 0.5 mM dithionite, and 4.4 µM tRNA.

### Microscale thermophoresis

Purified and reconstituted His-tagged *Min* Elp3 or *Min* Elp3 variants were incubated with iFluor 647-Tris NTA dye (12618, AAT Bioquest; final concentrations 40 nM dye and 50 nM protein) for 30 min in an anaerobic chamber in MST buffer (reaction buffer with 0.05% Tween 20) to achieve a degree-of-labelling of 0.8. The dye-labelled protein was spun down at 14,000 x *g* for 10 min to eliminate any aggregates from solution, then added in a 1:1 ratio with varying concentrations of *S*-adenosyl-*L*-methionine (final protein concentration of 50 nM, final SAM concentrations ranging from 0.054 – 4.5 mM). The samples were applied to MST capillaries (MO-K022, Nanotemper Technologies), which were then sealed on both ends with capillary wax (HR4-328, Hampton Research) to maintain an anaerobic environment during MST experiments. Measurements of triplicate samples from distinct capillary tubes were performed on a Monolith NT.115 with 20% excitation power and 40% MST power at room temperature. The data obtained were transformed from relative fluorescence to fraction bound and fit to a hill binding equation to determine the dissociation constant (*K*_D_).

### Electrophoretic mobility shift assays (EMSAs)

EMSAs were carried out similarly to a previously published procedure for Elp3^76^, with some modifications. 250 nM tRNA was incubated with a 1:2 serial dilution of 20 µM to 39 nM Elp3, plus 0 µM Elp3 control, in incubation buffer (100 mM HEPES, pH 7.5, 750 mM NaCl, 25 mM DTT) at 20 °C for 30 min. Non-denaturing 5% TBE gels (BioRad) were pre-run in running buffer (3.3 g Tris, 14.4 g glycine, 150 mg DTT per L) at 5 mA for 1 h at 4 °C. 10 µL of the incubated Elp3-tRNA samples were mixed with 2 µL of 50% glycerol in incubation buffer and loaded onto the gel. Gels were run at 5 mA for ~2 h with fresh running buffer, stained with Sybr Gold (Thermo Fisher Scientific) for 30 min, and imaged (Cell Biosciences FluorChem Q). Gel images were analyzed using ImageJ^77^. EMSAs were carried out in triplicate from separate samples.

### Acetyl-CoA hydrolysis assays

In an anaerobic chamber, 2X concentration solutions of Elp3 and AcCoA were incubated at 16 °C for 10 min in 2X reaction buffer, then mixed with a 2X concentration solution of tRNA to initiate AcCoA hydrolysis at a final reaction concentration of 10 µM Elp3, 100 µM AcCoA, and 2 µM tRNA. Reactions were then incubated at 37 °C for 30 min and quenched by freezing. AcCoA consumed in each reaction tube was determined using fluorescence-based AcCoA/CoA assay kits (Sigma MAK039, or Abcam ab102504) and measured on a TECAN Spark plate reader. All reported AcCoA hydrolysis assays were carried out in triplicate, measured from separate samples.

### Reporting summary

Further information on research design is available in the Nature Portfolio Reporting Summary linked to this article.

## Supplementary information

Supplementary Information

Reporting Summary

Transparent Peer Review file

## Source data

Source Data

## Data Availability

All biochemical data generated in this study are provided in the Source Data file. Any other data that support the findings of this study are available from the corresponding author upon request. PDB accession codes used in this study: 8ASW, 6IA6, 6IA8, 8PTX. Source data are provided with this paper.
