# Supplementary material for: Elp3 uses a conserved molecular tunnel to transport acetate between distant active sites and catalyze tRNA wobble base modification
Source: Nat Commun. 2026 Jun 3;17:5633. doi: 10.1038/s41467-026-73699-5 (PMC13315578; doi:10.1038/s41467-026-73699-5)
Supplement: Supplementary file 1 — Supplementary Information [file 41467_2026_73699_MOESM1_ESM.pdf]

Supplementary Information

Elp3 uses a conserved molecular tunnel to transport acetate between distant active sites and catalyze tRNA wobble base modification

Evan P. Geissler, Youmna Moawad, Paige N. Roehling, Cassidy Driscoll, Katherine Martin, Papa Nii Asare-Okai, Jeffrey S. Mugridge*

*Corresponding author. Department of Chemistry & Biochemistry, University of Delaware, Newark, DE 19716, United States; Email: mugridge@udel.edu

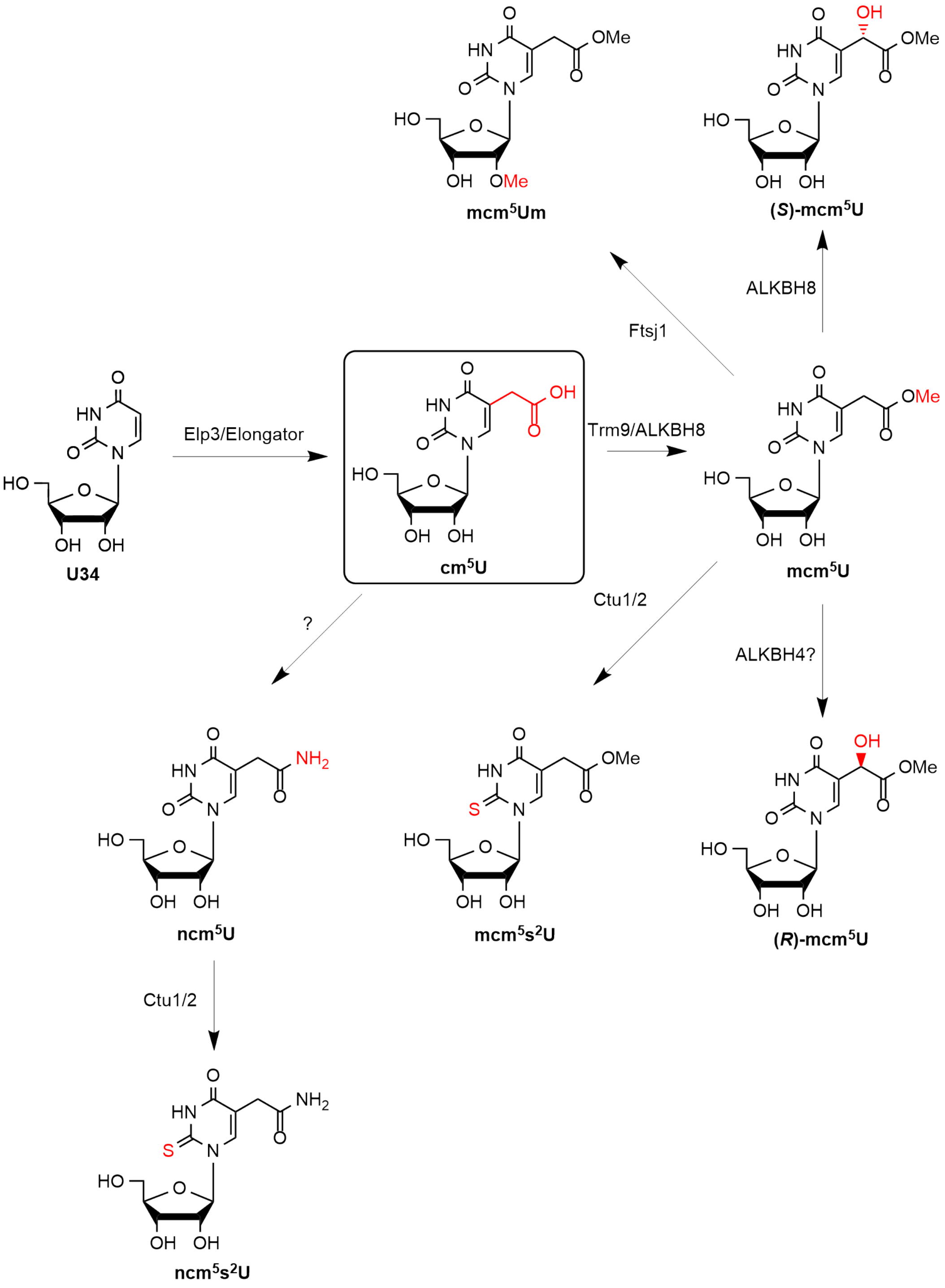

Supplementary Figure 1. 5-carboxymethyluridine (cm^5U)-derived eukaryotic tRNA modifications and modification enzymes. Elp3 and Elongator install the central intermediate tRNA modification cm^5U at the wobble base position (U34). cm^5U is further elaborated by additional tRNA modification enzymes to produce a family of cm^5U-derived modifications that impact translation efficiency and fidelity. Eukaryotic cm^5U-derived modifications and their known or speculated tRNA modification enzymes are shown.

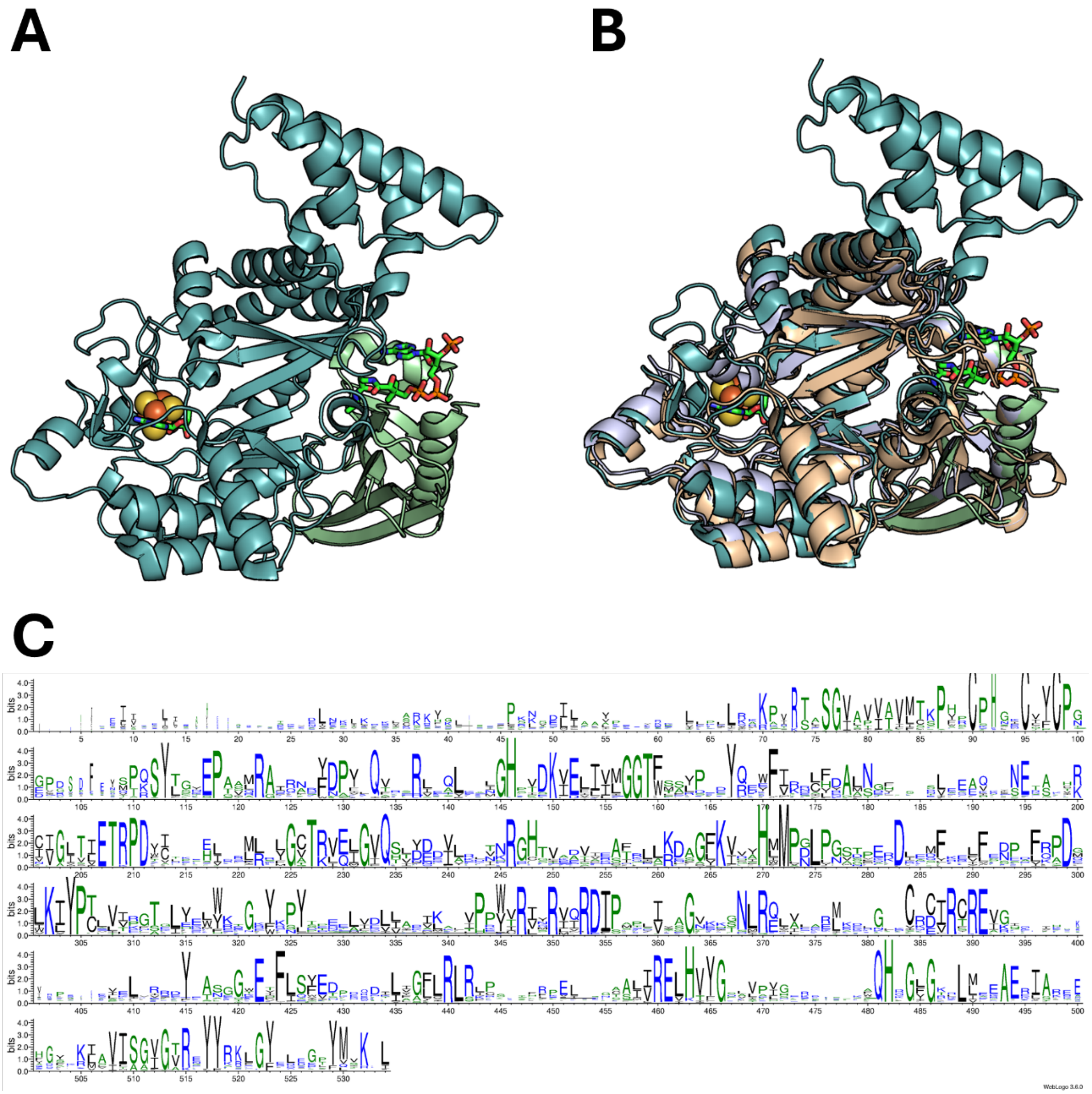

Supplementary Figure 2. Elp3 has a highly conserved sequence and tertiary structure. (A) Structure of *Saccharomyces cerevisiae* Elp3 (rSAM domain in teal and KAT domain in light green, PDB 8ASW) with 5′-dA and AcCoA analogue desulfo-CoA (aligned from PDB 6IA6) shown as green sticks. **(B)** Elp3 structural alignment from *S. cerevisiae* (eukaryote, rSAM domain in teal and KAT domain in light green, PDB 8ASW), *Dehalococcoides mccartyi* (bacteria, light blue, PDB 6IA6), and *Methanocaldococcus infernus* (*Min*, archaea, tan, PDB 6IA8). 5′-dA (PDB 8ASW) and AcCoA analog desulfo-CoA (PDB 6IA6) are shown as green sticks and the [4Fe-4S] cluster (PDB 8ASW) is shown in orange and yellow spheres. **(C)** Elp3 sequence alignment using DeepMSA2[1] with an alignment depth (Nf) of 18.03 from 568 sequences spanning archaea, bacteria, and both lower and higher eukaryotes. Visualized with Weblogo.[2] Residues are numbered according to the *Min* sequence.

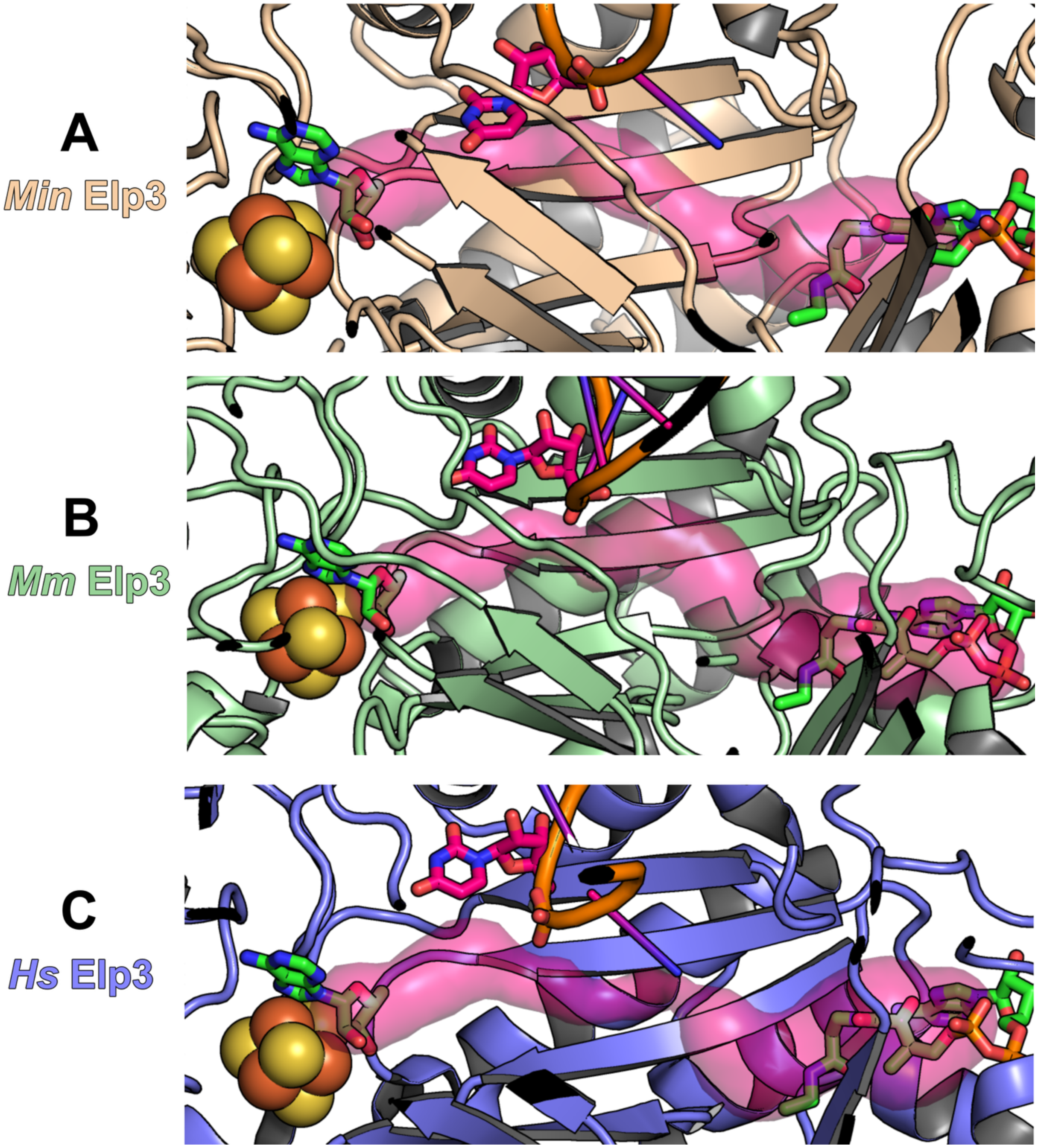

Supplementary Figure 3. A molecular tunnel connecting rSAM and KAT sites is conserved in Elp3-tRNA complexes across all domains of life. CAVER analyses were performed on Elp3-tRNA models of **(A)** *Methanocaldococcus infernus* Elp3 (tan; AlphaFold3-generated model with tRNA Arg^{UCU} anticodon stemloop), **(B)** *Mus musculus* Elp3 (light green; AlphaFold3-generated model with 15-mer tRNA Lys^{UUU} anticodon stemloop), and **(C)** *Homo sapiens* Elp3 (blue; from cryo-EM-determined structure 8PTX with bound tRNA Gln^{UUG}). In **A** – **C**, substrate tRNA base U34 is shown as hot pink sticks, [4Fe-4S] cluster is shown as yellow and orange spheres, and 5′-dA and desulfo-CoA are

shown as green sticks; [4Fe-4S] cluster and 5′-dA were modeled from PDB 8PTX and desulfo-CoA was modeled from PDB 6IA6. Like the yeast Elp3-tRNA complex, all of these models reveal similar enclosed molecular tunnels that travel across the Elp3-tRNA complex, connecting the KAT domain AcCoA binding site to the [4Fe-4S]-containing rSAM active site. These CAVER analyses spanning archaeal, yeast, and mammalian species suggest that the molecular tunnel is conserved in Elp3-tRNA complexes across all domains of life and supports our proposed mechanism of Elp3-mediated tRNA modification in which acetate molecules are delivered to the rSAM site via transport through the molecular tunnel.

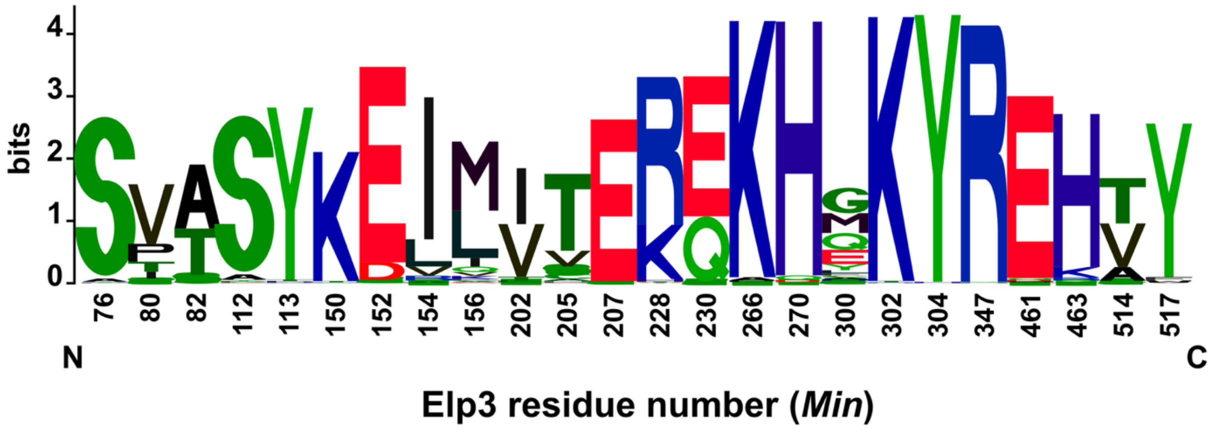

Supplementary Figure 4. The residues forming Elp3's molecular tunnel are highly conserved. Tunnel-lining residues whose sidechain compose part of the tunnel surface are shown and numbered according to the *Min* sequence. Alignment generated with DeepMSA2[1] (full alignment shown in Figure S2, Nf = 18.03, 568 sequences spanning archaea, bacteria, and eukaryotes) and visualized with LogoJS.[3]

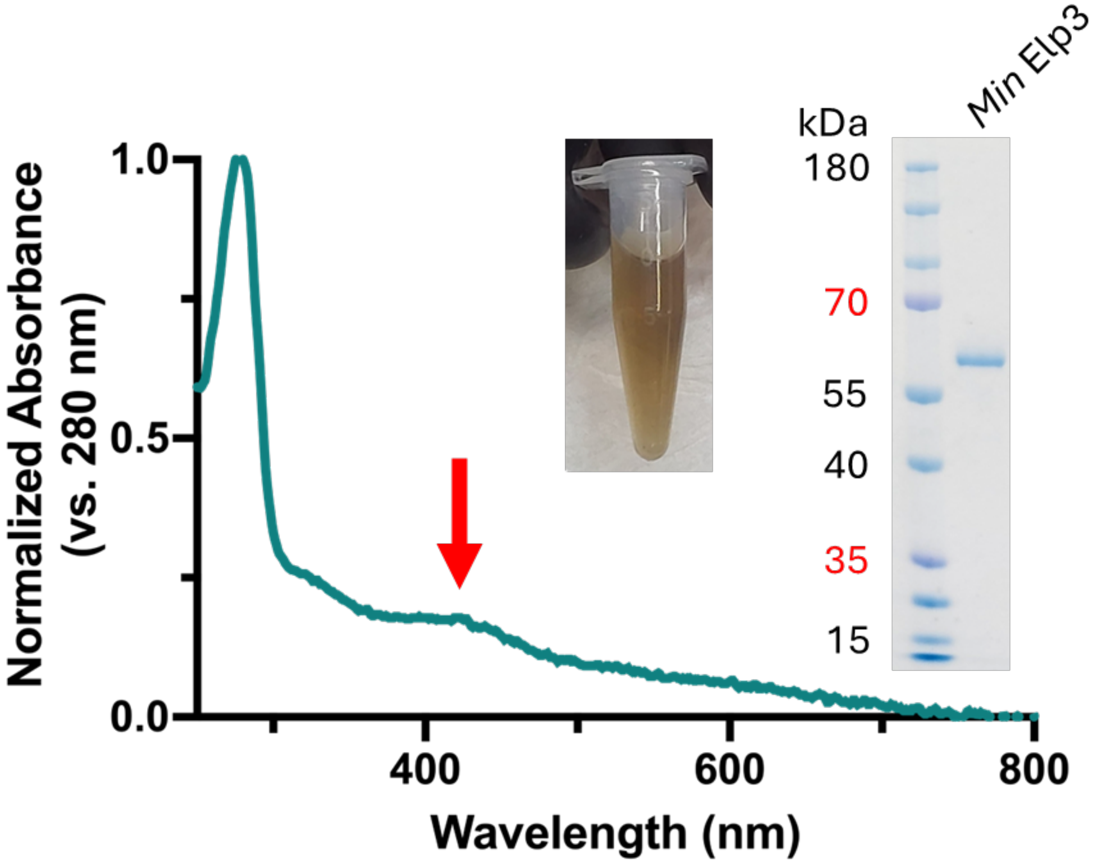

Supplementary Figure 5. Characterization of WT *Min* Elp3. The UV-Vis spectrum of purified, reconstituted *Min* Elp3 has a shoulder at 420 nm (red arrow) consistent with the presence of a [4Fe-4S] cluster. The inset images show the characteristic brown-yellow color of *Min* Elp3 following reconstitution and its purity as assessed by gel electrophoresis. The *Min* Elp3 construct is predicted to have a mass of 65.0 kDa.

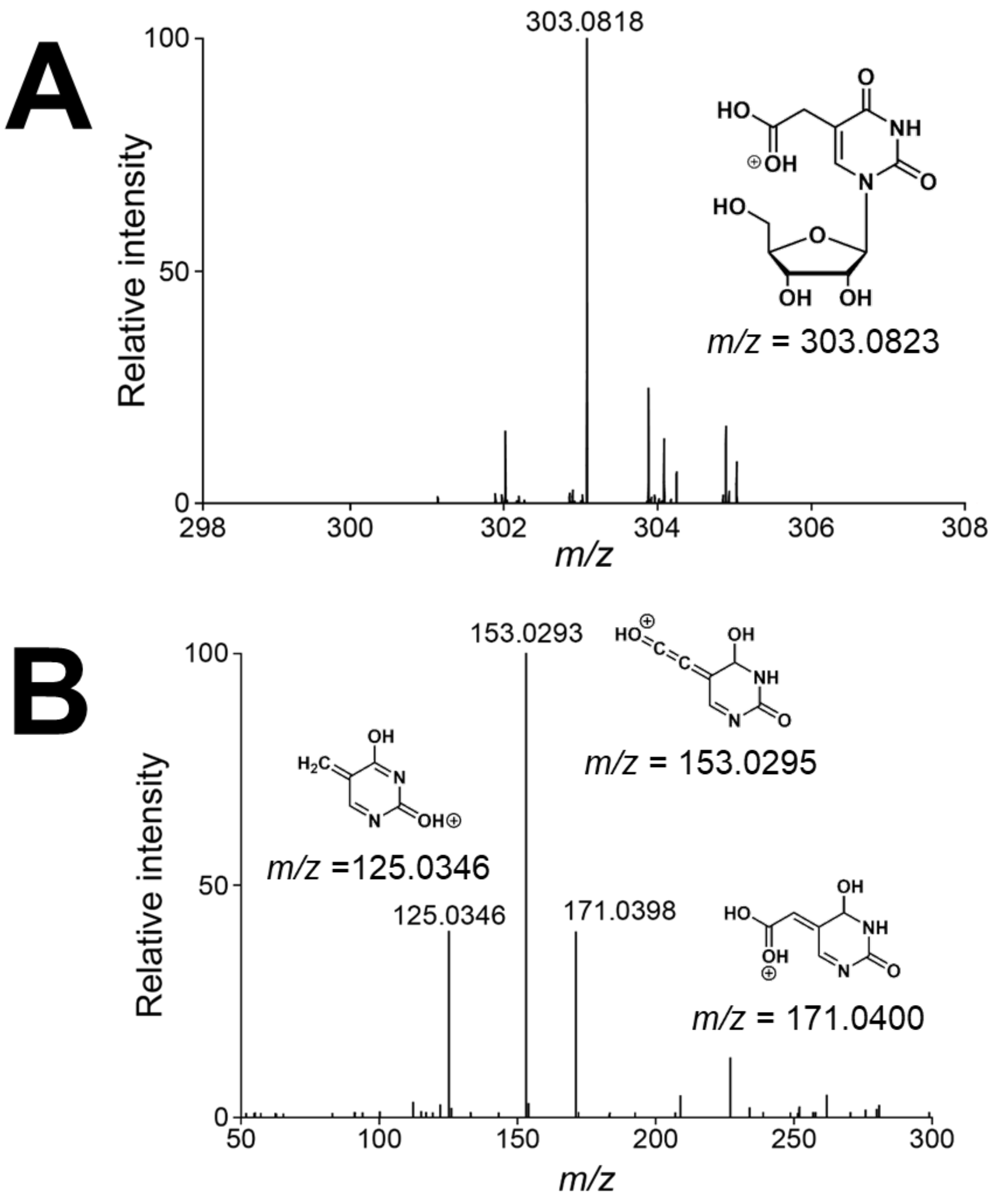

Supplementary Figure 6. Mass spectra of cm^{5}U product from Elp3 reactions. (A) LC-MS analysis of nuclease-digested tRNA nucleosides confirms that Elp3 reactions with non-isotopically labeled AcCoA produce cm^{5}U. **(B)** MS/MS analysis of the cm^{5}U nucleoside product peak revealed characteristic mass fragments of cm^{5}U (compared to a commercial cm^{5}U standard). The tRNA modification reaction was performed with 5 μM Elp3, 4.4 μM tRNA, 25 μM SAM, 0.5 mM dithionite, and 27.5 μM AcCoA. MS ion structures and masses were predicted with CFM-ID 4.0.[4]

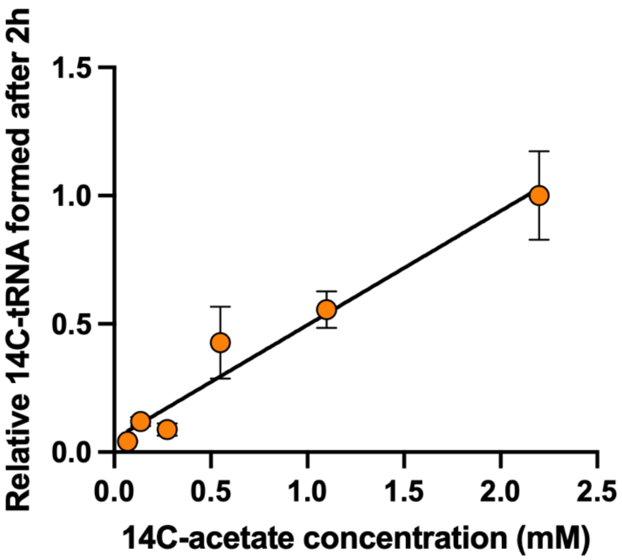

Supplementary Figure 7. Amount of product ^{14}C-tRNA formed during Elp3 reactions is linearly dependent on ^{14}C-acetate concentration. Incubation of *Min* Elp3 with substrate tRNA Arg^{UCU} and increasing concentrations of ^{14}C-acetate for 2 hour reaction times results in linearly increasing amounts of ^{14}C-labeled tRNA product formation. Activity assays were performed in triplicate with 5 µM Elp3, 4.4 µM tRNA, 25 µM SAM, 0.5 mM dithionite as reductant, and 2-fold dilutions of ^{14}C-acetate concentrations from a max concentration of 2.2 mM. A linear fit of the activity data is shown in black, with errors shown as mean values ± SEM (n = 3).

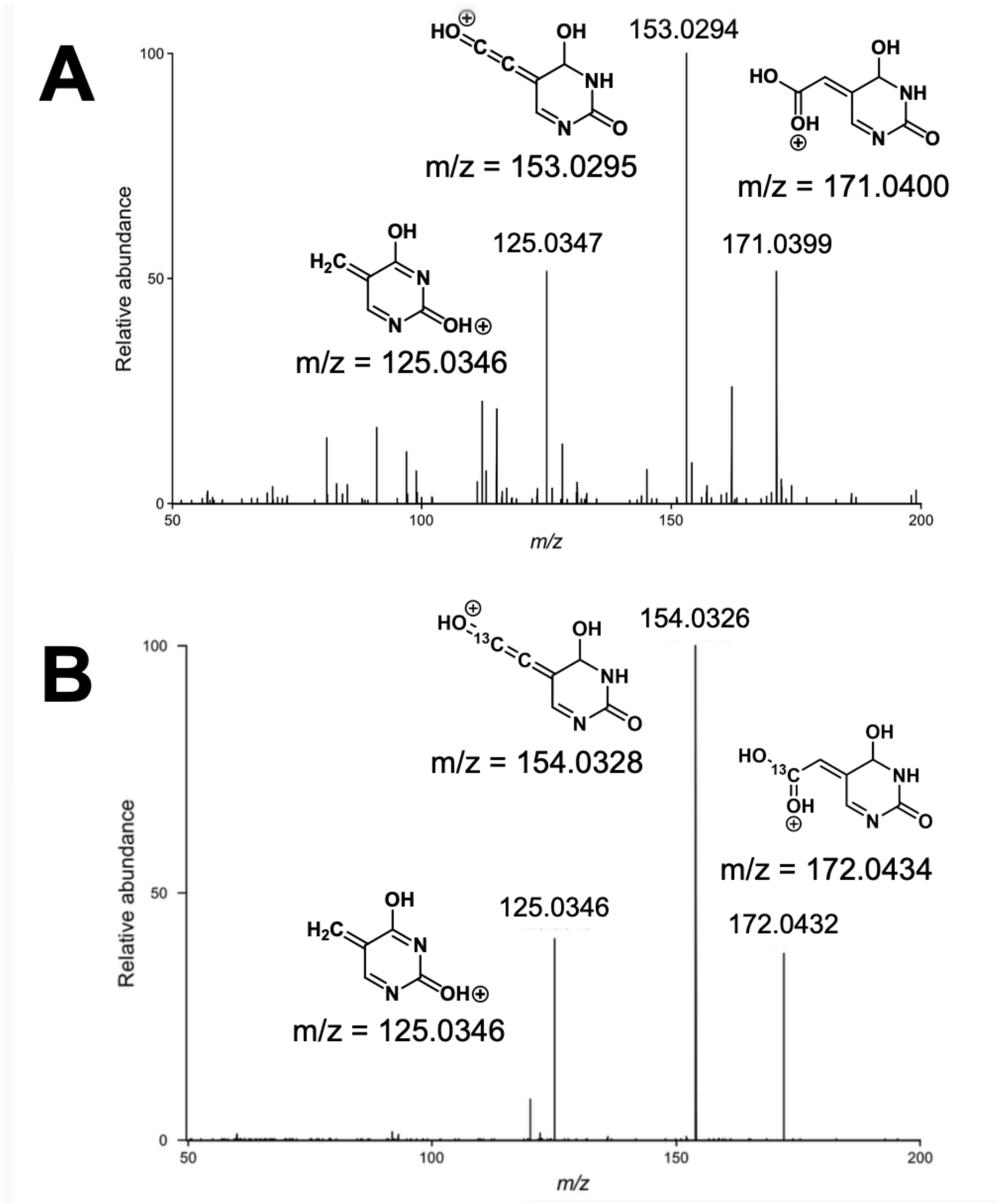

Supplementary Figure 8. MS/MS spectra of cm^{5}U product from (A) ^{12}C and (B) ^{13}C acetate reactions. (A) MS/MS analysis of the ^{12}C (non-isotopically labeled) acetate reaction cm^{5}U nucleoside product peak revealed characteristic mass fragments of cm^{5}U. **(B)** MS/MS analysis of the ^{13}C acetate reaction cm^{5}U nucleoside product peak revealed characteristic mass fragments of cm^{5}U with appropriate +1 Da mass shifts based on the location of the ^{13}C label. tRNA modification reactions were performed with 5 μM Elp3, 4.4 μM tRNA, 25 μM SAM, 0.5 mM dithionite, and 10 mM ^{12}C or ^{13}C acetate. MS ion structures and masses were predicted with CFM-ID 4.0.[4]

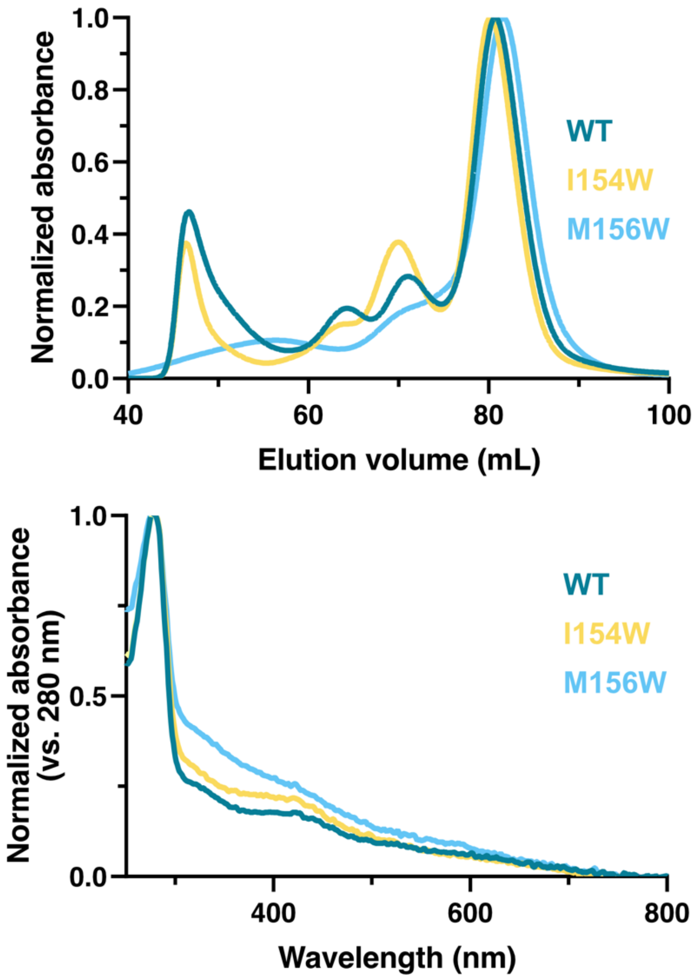

Supplementary Figure 9. Comparison of purified and reconstituted M156W and I154W vs WT *Min* Elp3. (A) Normalized size exclusion chromatography (SEC) traces of WT (teal), M156W (blue) and I154W (yellow) *Min* Elp3. **(B)** UV-Vis spectra of WT (teal), M156W (blue) and I154W (yellow) *Min* Elp3 after reconstitution; all spectra show similar, characteristic [4Fe-4S] absorbance shoulders at 420 nm.

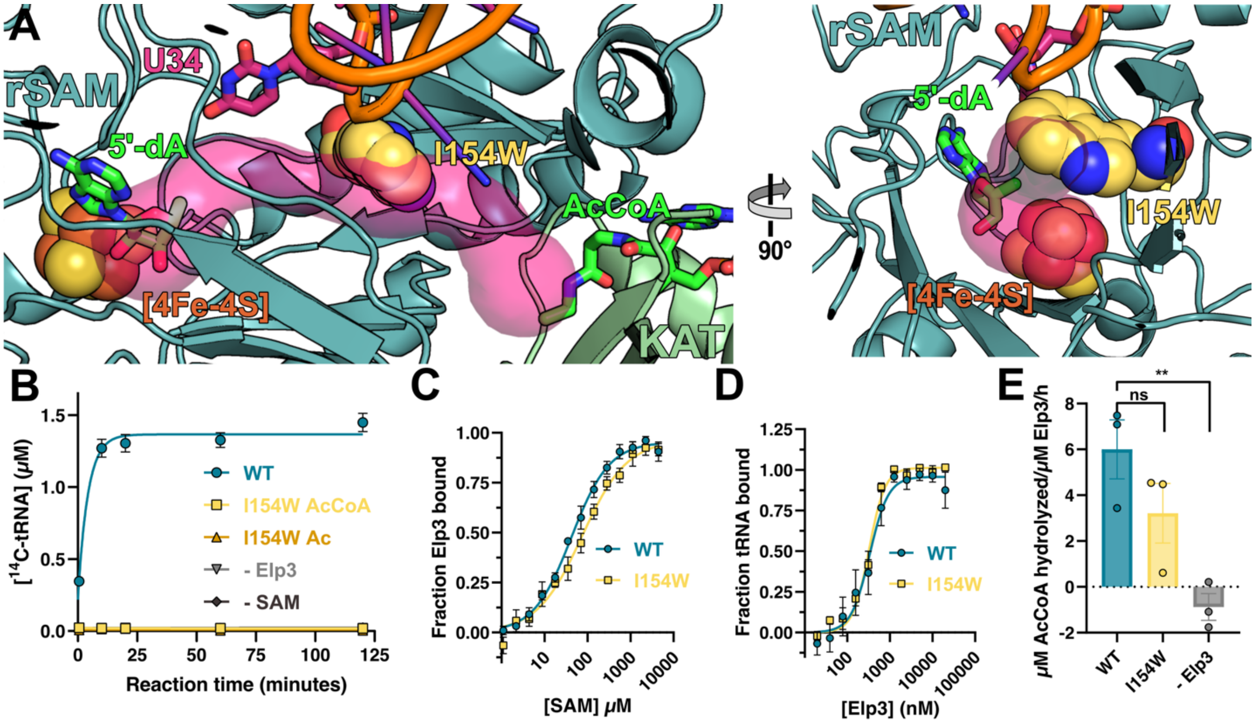

Supplementary Figure 10. **The Elp3 I154W mutation blocks the molecular tunnel and eliminates tRNA-modifying activity without significantly disrupting SAM binding, tRNA binding, or AcCoA hydrolysis. (A)** In addition to the M156W mutation shown in Figure 2, mutagenesis modeling and analysis of Elp3 bound to tRNA (PDB 8ASW) suggested that the I154W mutation (shown in yellow spheres), located midway along the molecular tunnel, will substantially block Elp3's tunnel and likely impede diffusion of acetate from the KAT AcCoA binding site to the rSAM active site. **(B)** Incubation of Elp3 I154W with substrate tRNA Arg^{UCU} and ^{14}C-labeled AcCoA (yellow squares) or ^{14}C-labeled acetate (orange triangles) *in vitro* results in no observable tRNA modification activity after 2 hours compared to WT *Min* Elp3 (teal circles); activity of the I154W mutant is comparable to -Elp3 (gray triangles) and -SAM (dark brown diamonds) negative controls. Triplicate time courses were performed with 5 μM Elp3, 4.4 μM tRNA, 25 μM SAM, 0.5 mM dithionite as reductant, and either 27.5 μM ^{14}C-AcCoA or 2 mM ^{14}C-acetate, with data fit to a single-phase exponential equation and errors shown as mean values ± SD (n = 3). **(C)** Elp3 I154W (yellow squares) binds cofactor SAM with comparable affinity to WT Elp3 (teal circles). Elp3-SAM binding was measured by triplicate microscale thermophoresis (MST) assays, and fraction bound data were fit to a hill binding model with errors shown as mean values ± SEM (n = 3). **(D)** Elp3 I154W (yellow squares) binds substrate tRNA Arg^{UCU} with comparable affinity to WT Elp3 (teal circles). Elp3-tRNA binding was measured by triplicate electrophoretic mobility shift assays (EMSAs; **Figure S12**), and fraction bound data were fit to a hill binding model with errors shown as mean values ± SEM (n = 3). **(E)** Elp3 I154W (yellow) may moderately reduce AcCoA hydrolysis activity in the presence of substrate tRNA Arg^{UCU} compared to WT Elp3 (teal), but the

measured differences were not statistically significant; both WT and I154W Elp3 show more AcCoA hydrolysis activity than a -Elp3 negative control (gray). AcCoA hydrolysis was measured in triplicate with a commercial fluorometric AcCoA/CoA quantification kit and statistical significance was calculated using a one-way ANOVA with Dunnett's multiple comparisons test (** $p \leq 0.01$, ns $p > 0.05$); errors shown as mean values ± SEM.

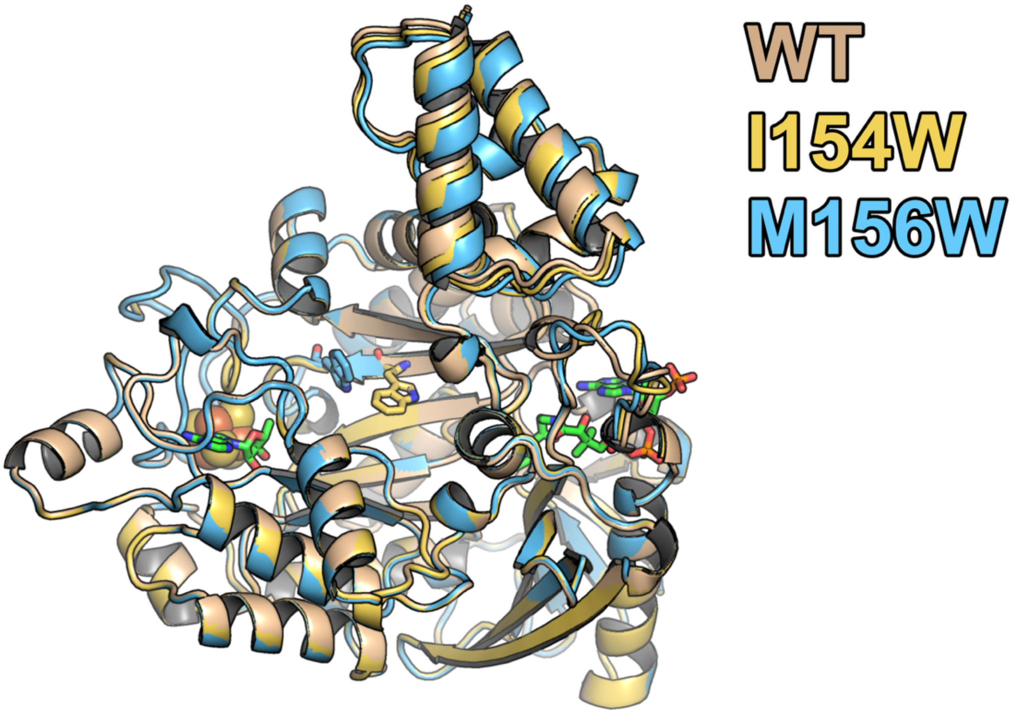

Supplementary Figure 11. AlphaFold3 models suggest tunnel-blocking mutations do not affect the Elp3 fold. Tunnel blocking mutations I154W (yellow) and M156W (blue) have nearly identical folds to WT Elp3 (tan), according to AlphaFold3 predictions and alignment in PyMOL (RMSD < 0.2 relative to WT Elp3). The location of mutated Trp sidechains are shown as sticks. The similar predicted fold for these Elp3 variants is consistent with our biochemical experiments showing M156W and I154W mutations do not significantly disrupt tRNA binding, SAM binding, or AcCoA turnover. After AlphaFold3 prediction, [4Fe-4S] cluster (yellow/orange spheres) and 5′-dA (green sticks) were modeled in from PDB 8PTX and desulfo-CoA (green sticks) was modeled from PDB 6IA6, for visualization.

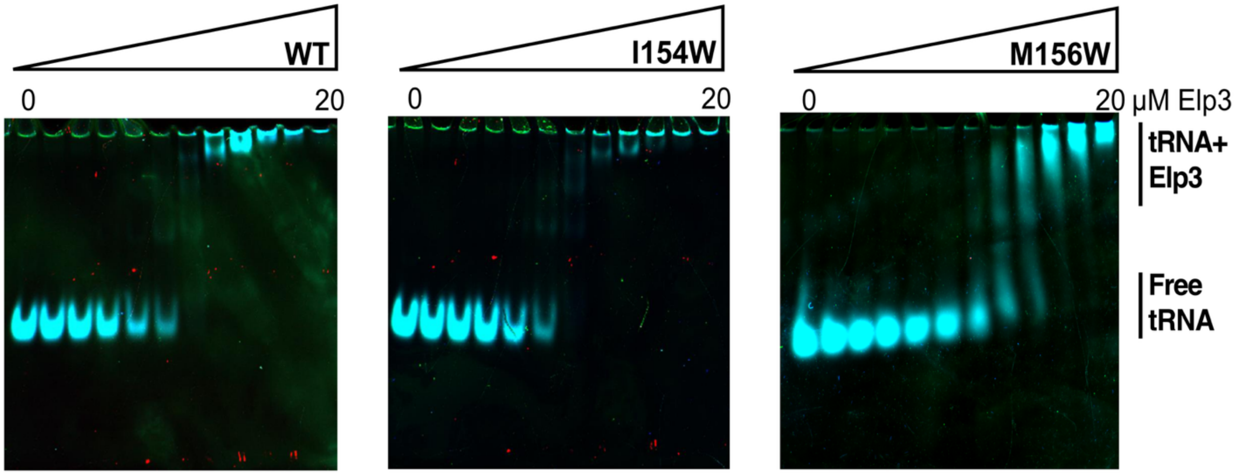

Supplementary Figure 12. Representative EMSA gels used to measure tRNA binding to Elp3 tunnel blocking mutants. 250 nM tRNA was incubated with 0 – 20 µM Elp3, free and bound tRNA species were separated on a 5% TBE gel, and tRNA was visualized with SYBR gold staining (stained tRNA is cyan). Elp3-tRNA complexes migrate very slowly on the gel and were difficult to reliably quantify, so fraction bound was calculated by quantifying the amount of free tRNA at each Elp3 concentration compared to total tRNA (0 µM Elp3).

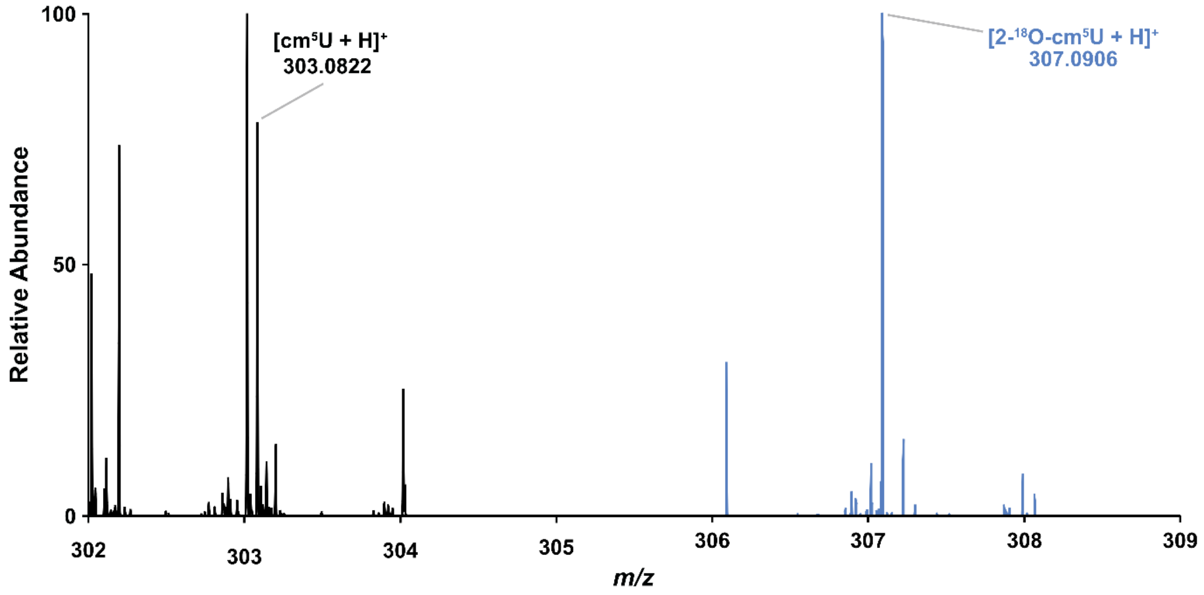

Supplementary Figure 13. Uncropped mass spectra of the ^{12}C and $^{18}O_2$ acetate cm^{5}U nucleoside products. The black LC-MS spectrum shows the cm^{5}U product from ^{12}C-acetate reactions with Elp3; the blue LC-MS spectrum shows the cm^{5}U product from $^{18}O_2$-acetate reactions with Elp3, showing the +4 Da shift from the isotopically unlabeled acetate reactions. This is the same data as shown in Figure 3B, but with an uncropped x-axis.

A

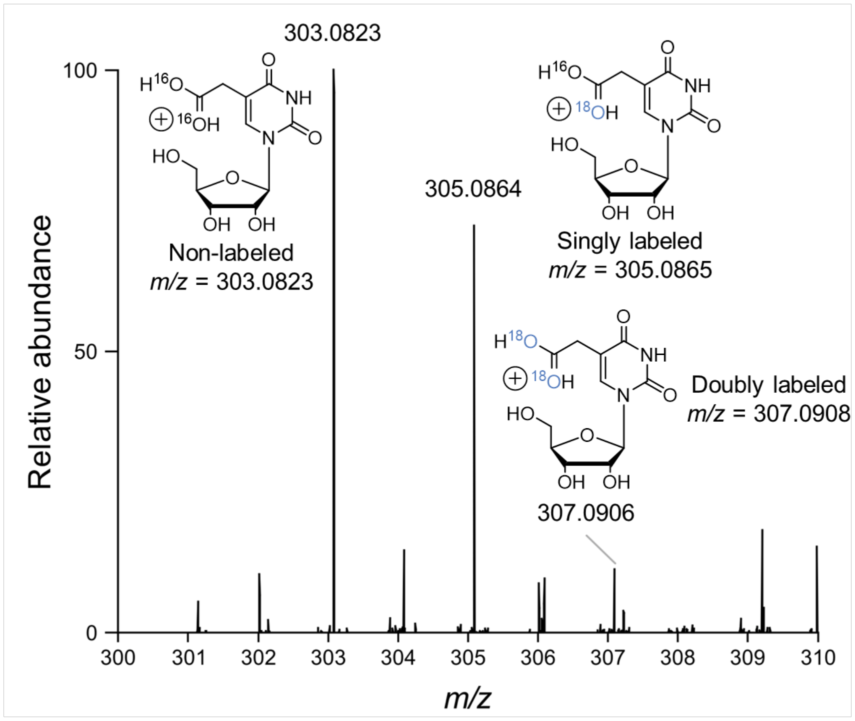

B

(i) **Isotope ratio relative to ^{16}O cm^{5}U**

Ratio	0-^{18}O (all ^{16}O)	1-^{18}O (+2 Da)	2-^{18}O (+4 Da)
Theoretical	99.91	1.71	0.01
Observed	100	71.47	10.44

(ii) **Isotope ratio relative to singly-labeled ^{18}O cm^{5}U**

Ratio	1-^{18}O (+2 Da)	2-^{18}O (+4 Da)
Theoretical	99.97	1.51
Observed	100	11.70

Supplementary Figure 14. ^{18}O ratio analysis for Elp3-mediated reactions with $^{18}O_2$-acetate. (A) LC-MS analysis of the *in vitro* activity assay with $^{18}O_2$ acetate showing doubly and singly ^{18}O-labeled cm^{5}U, as well as nonlabelled cm^{5}U. The cm^{5}U [M+H] masses were predicted with enviPat.[5] **(B)** An analysis of the oxygen isotope ratios between different isotopically labeled cm^{5}U products reveals that doubly ^{18}O-labeled cm^{5}U is observed at a relative abundance **(i)** ~1000-fold higher than would be expected in normal abundance (theoretical) cm^{5}U and **(ii)** ~8-fold higher than would be expected in a singly ^{18}O-labeled-only cm^{5}U sample. Theoretical ratios expected for the specific LC-MS instrument used were predicted with enviPat (resolution setting: Q-Exactive, ExactivePlus_R140000@200`) and observed ratios were calculated using the peak areas of the various cm^{5}U isotopes.

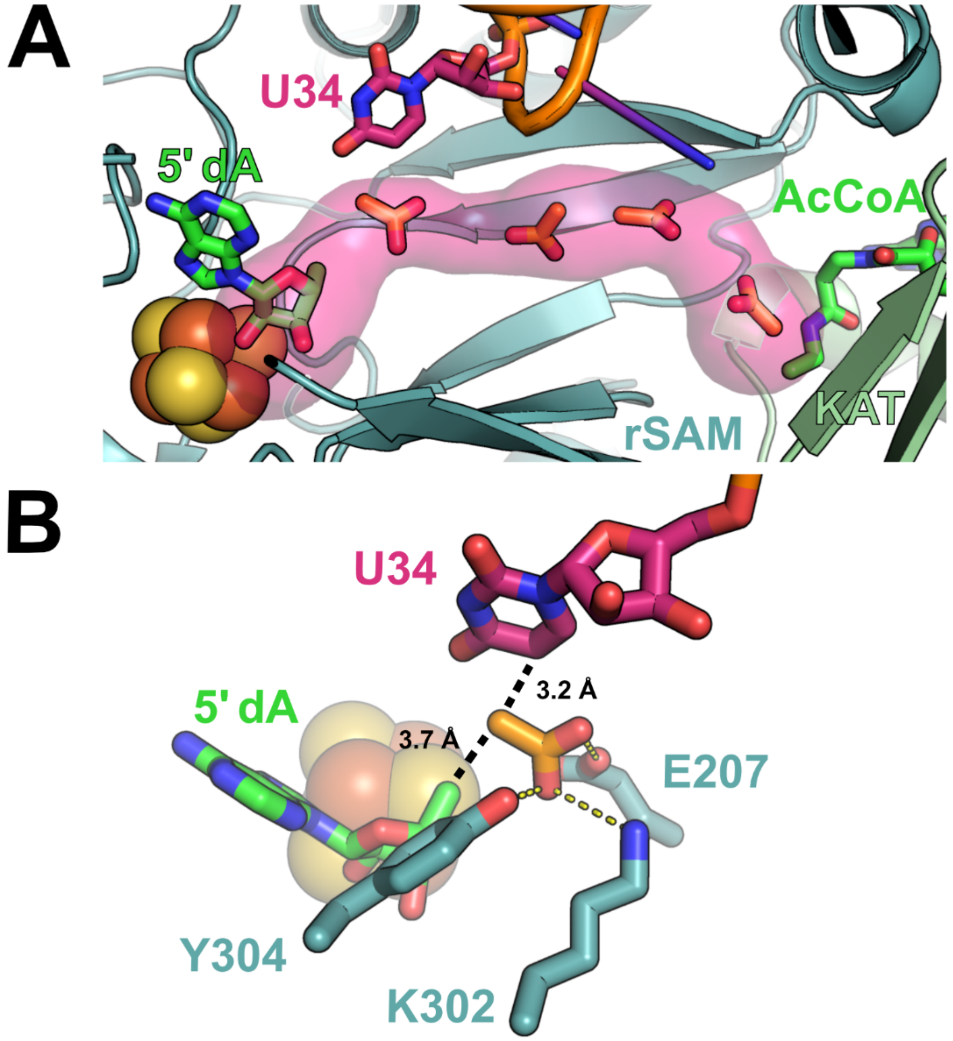

Supplementary Figure 15. Molecular docking provides hypotheses about how acetate may occupy the Elp3 molecular tunnel. (A) Docking acetate in the yeast structure of Elp3 bound to tRNA (PDB 8ASW) with CaverDock[6] suggests that acetate can reasonably be accommodated at many positions along the molecular tunnel. The Elp3 rSAM domain is colored teal, the Elp3 KAT domain is colored light green, the calculated tunnel is shown in pink, and 4 CaverDock-predicted acetate poses are shown in orange sticks along the length of the tunnel; 5′-dA and AcCoA analog desulfo-CoA (aligned from PDB 6IA6) are shown in green sticks; tRNA and substrate tRNA base U34 are shown in orange and pink, respectively. **(B)** CaverDock modeling of acetate in the rSAM active site. The CaverDock pose shown here provides a similar, but alternative, conformation of acetate in the Elp3 active site, as compared to the SeamDock model shown in Figure 4A. Both this model and the one in Figure 4A show how acetate may be oriented by noncovalent interactions (yellow dotted lines) in the rSAM active site that position the acetate methyl group at a reasonable distance for sequential reaction with 5′-dA· and tRNA U34.

Min	Yeast	Human
E207	E230	E221
K302	K325	K316
Y304	Y327	Y318

Supplementary Table 1. Numbering of key, conserved rSAM active site residues for archaeal *Methanocaldococcus infernus* (*Min*), yeast, and human Elp3.

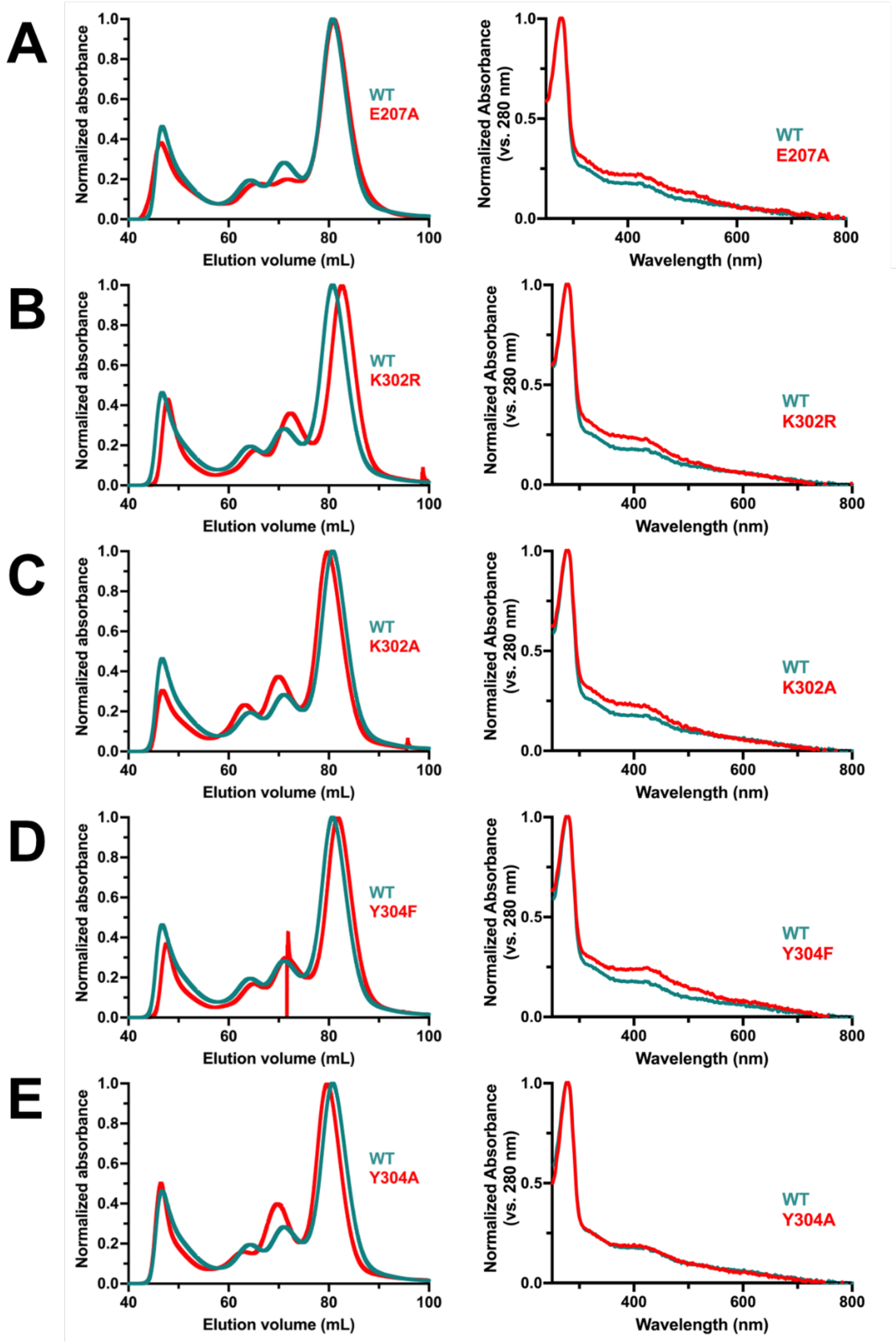

Supplementary Figure 16. Comparison of the purified and reconstituted rSAM active site variants to WT *Min* Elp3. SEC traces (left) and UV-Vis spectra (right) of the rSAM site variants E207A **(A)**, K302R **(B)**, K302A **(C)**, Y304F **(D)**, and Y304A **(E)** compared to WT Elp3. All Elp3 mutants show similar SEC traces and similar, characteristic [4Fe-4S] absorbance peaks at 420 nm, as compared to WT *Min* Elp3.

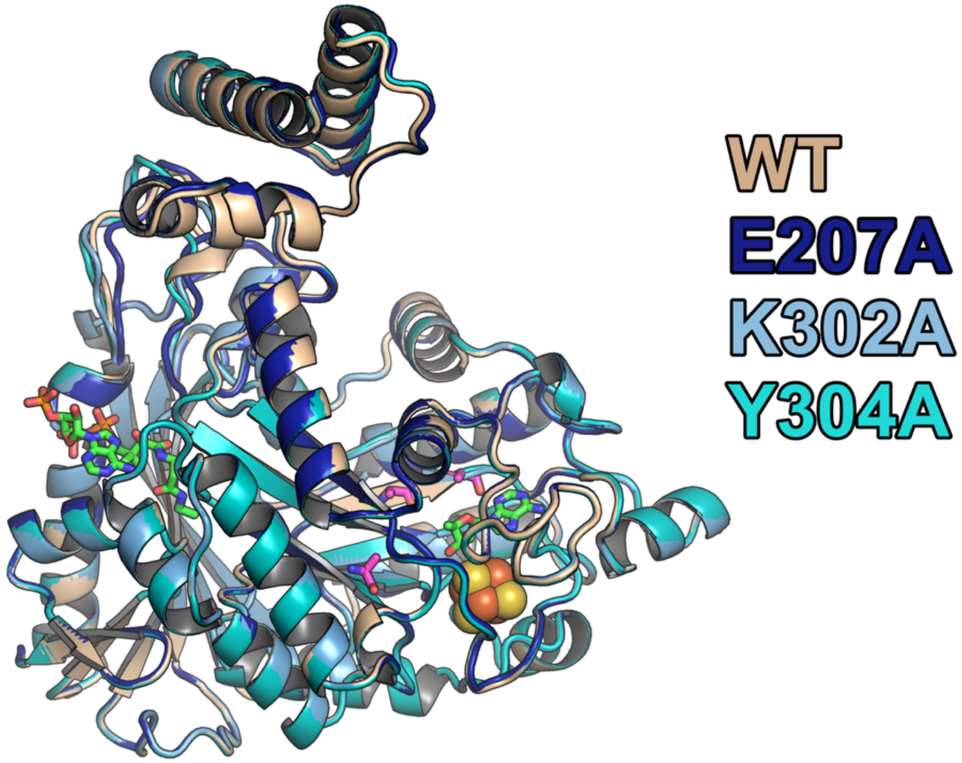

Supplementary Figure 17. AlphaFold3 models suggest mutations to conserved tunnel-lining / active site residues do not affect the Elp3 fold. Conserved tunnel-lining rSAM active site Elp3 mutants E207A (dark blue), K302A (light blue), and Y304A (cyan) have nearly identical folds to WT Elp3 (tan), according to AlphaFold3 predictions and alignment in PyMOL (RMSD < 0.2 relative to WT Elp3). The location of mutated Ala sidechains are highlighted as pink sticks. After AlphaFold3 prediction, [4Fe-4S] cluster (yellow/orange spheres) and 5′-dA (green sticks) were modeled in from PDB 8PTX and desulfo-CoA (green sticks) was modeled from PDB 6IA6, for visualization.

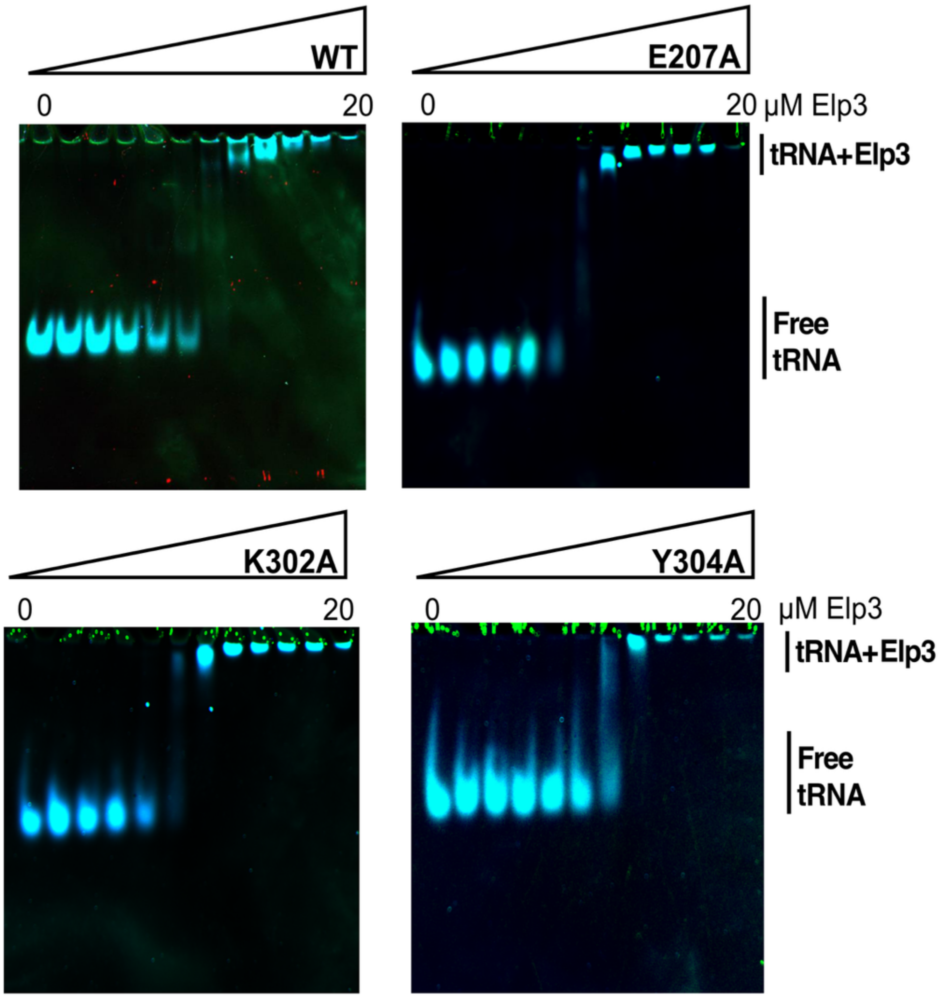

Supplementary Figure 18. Representative EMSA gels used to measure tRNA binding to Elp3 rSAM active site mutants E207A, K302A, and Y304A. 250 nM tRNA was incubated with 0 – 20 µM Elp3, free and bound tRNA species were separated on a 5% TBE gel, and tRNA was visualized with SYBR gold staining (stained tRNA is cyan). Elp3-tRNA complexes migrate very slowly on the gel and were difficult to reliably quantify, so fraction bound was calculated by quantifying the amount of free tRNA at each Elp3 concentration compared to total tRNA (0 µM Elp3).

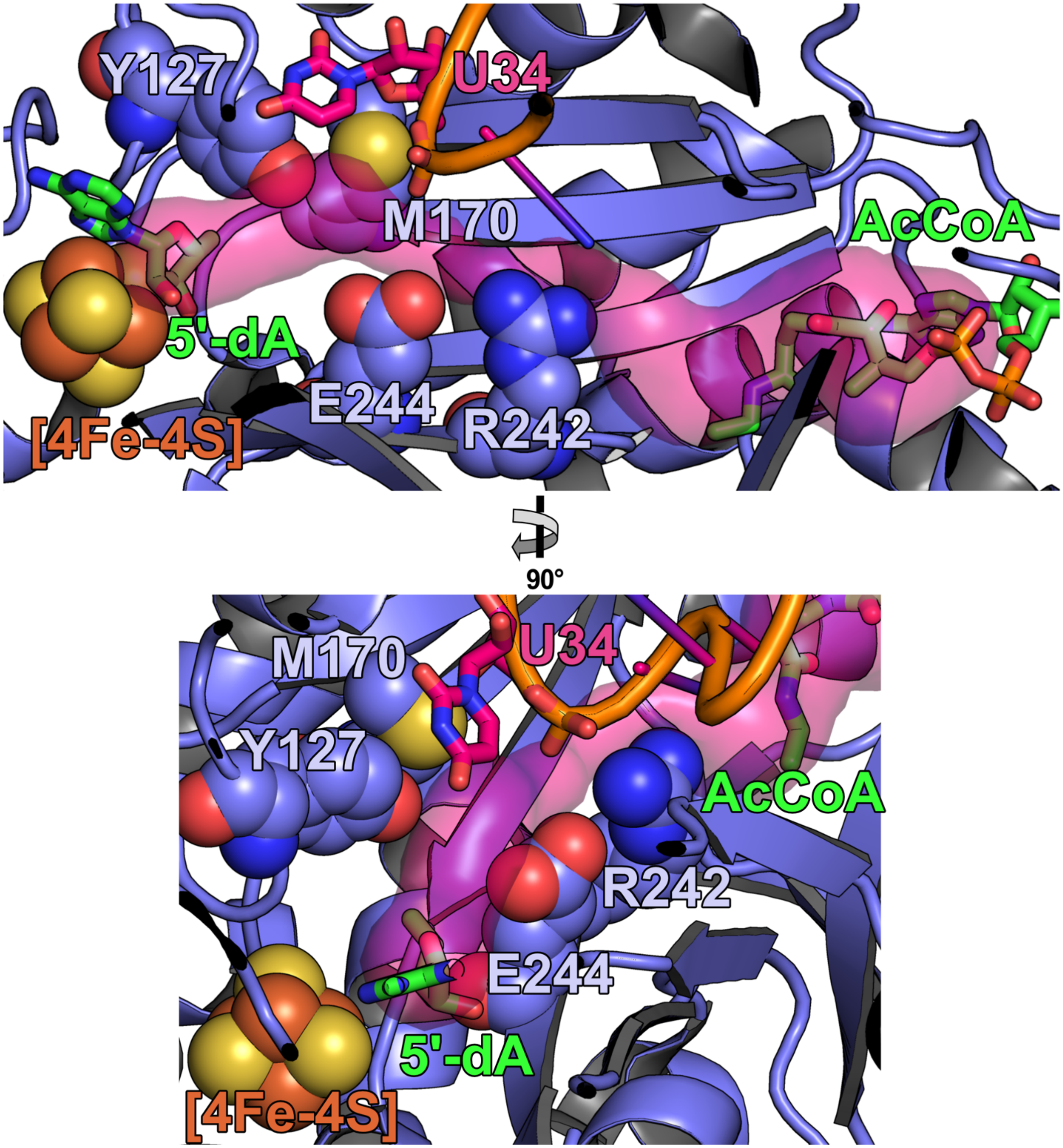

Supplementary Figure 19. Tunnel-lining residue mutations identified in tumor sequencing databases. *Homo sapiens* Elp3 structure (blue; from cryo-EM-determined structure 8PTX with bound tRNA Gln^{UUG}) with CAVER-calculated molecular tunnel in pink, cancer-related residues Y127, M170, R242, and E244 shown as spheres, substrate tRNA base U34 shown as hot pink sticks, and 5′-dA and desulfo-CoA (from PDB 6IA6) are shown as green sticks.

Supplementary References

1. Zheng, W.; Wuyun, Q.; Li, Y.; Zhang, C.; Freddolino, P. L.; Zhang, Y. Improving deep learning protein monomer and complex structure prediction using DeepMSA2 with huge metagenomics data. *Nat Methods* **2024***, 21*, 279–289.

2. Crooks, G. E.; Hon, G.; Chandonia, J.; Brenner, S. E. WebLogo: A Sequence Logo Generator. *Genome Res.* **2004***, 14*, 1188-1190.

3. Pratt, H. & Weng, Z. LogoJS: a Javascript package for creating sequence logos and embedding them in web applications. *Bioinformatics* **2020**, *36*, 3573–3575.

4. Wang, F.; Allen, D.; Tian, S.; Oler, E.; Gautam, V.; Greiner, R.; Metz, T. O.; Wishart, D. S. CFM-ID 4.0 – a web server for accurate MS-based metabolite identification. *Nucleic Acids Research* **2022***, 50*, W165–W174.

5. Loos, M.; Gerber, C.; Corona, F.; Hollender, J.; Singer, H. Accelerated Isotope Fine Structure Calculation Using Pruned Transition Trees. *Anal. Chem.* **2015***, 87*, 5738-5744.

6. Vavra, O.; Filipovic, J.; Plhak, J.; Bednar, D.; Marques, S. M.; Brezovsky, J.; Stourac, J.; Matyska, L.; Damborsky, J. CaverDock: a molecular docking-based tool to analyse ligand transport through protein tunnels and channels. *Bioinformatics* **2019***, 35*, 4986–4993.
