# Supplementary material for: Elp3 uses a conserved molecular tunnel to transport acetate between distant active sites and catalyze tRNA wobble base modification
Source: Nat Commun. 2026 Jun 3;17:5633. doi: 10.1038/s41467-026-73699-5 (PMC13315578; doi:10.1038/s41467-026-73699-5)
Supplement: Supplementary file 3 — Transparent Peer Review file [file 41467_2026_73699_MOESM3_ESM.pdf]

nature portfolio

Peer Review File

Elp3 uses a conserved molecular tunnel to transport acetate between distant active sites and catalyze tRNA wobble base modification

Corresponding Author: Professor Jeffrey Mugridge

This file contains all reviewer reports in order by version, followed by all author rebuttals in order by version.

Version 0:

Reviewer comments:

Reviewer #1

(Remarks to the Author)
Geissler et al. uncovered a novel catalytic mechanism of Elp3 forming Cm5U formation. Cm5U and its derivatives are widely conserved modification in eukaryotes and archaea. Lack of cm5U causes defects in cell fitness and involved in human diseases. Elp3 has two substrate binding sites, radical SAM motif and acetyl-CoA binding site. Previous reports showed that 5-dA radical generated at the radical SAM domain is activating acetyl group of acetyl-CoA, and then activated acetyl-CoA attack tRNA to form cm5U modification. However, since two reaction sites are far from each other, how 5-dA radical activates acetyl-CoA was outstanding question. In this manuscript, the authors conducted structural analysis using software that finds tunnels and channels in protein structure and found a molecular tunnel from Acetyl-CoA binding site to SAM binding site. Authors also discovered that cm5U formation is successfully conducted by acetate instead of acetyl-CoA. These observations support authors' proposed model that an acetate molecule released from acetyl-CoA travel through the molecular tunnel inside of Elp3 and activated at radical SAM domain and form the cm5U modification. The biochemical, mass spectrometric, and computational studies strongly support their reaction model. The manuscript is well written, experiments are appropriately designed and conducted, and their results robustly support authors' conclusion. The finding of unique catalytic mechanism underlying well-conserved and significant tRNA modification will be of great interest of the broad range of the readers of Nature Communications. This reviewer highly recommends publishing this paper.
One minor comment is that authors should discuss the conservation of tunnel structure among different Elp3 proteins. Authors found the tunnel in yeast Elp3 protein, but use the archaeal protein for the biochemical studies. I am wondering if the tunnel structure is also observed in archaeal homolog. If there is no available structure, checking a predicted structure of MinElp3 would be feasible to address this point

Reviewer #2

(Remarks to the Author)
In their manuscript the authors present new insights into the Elp3-catalyzed tRNA modification reaction, through the discovery of a tunnel that is used to shuttle the reaction intermediate acetate from the acetyl-CoA binding pocket to the radical SAM active site. In eukaryotes, including humans, Elp3 is the catalytic subunit of a large multi-protein complex, called Elongator consisting of the proteins Elp1-6, which modifies tRNA wobble uridines to 5-carboxymethyluridine (cm5U). Mutations in Elp3 and other Elongator subunits are linked to various diseases, including cancer and neurodegenerative and neurodevelopmental diseases. However, in many archaea and some bacteria, Elp3 exists as a stand-alone enzyme that conducts the same reaction. Due to the challenges in reconstituting the Elp3-catalyzed tRNA modification reaction, and despite the availability of structures of Elp3 and Elongator subcomplexes, an open question in the field that remained is how the SAM and AcCoA substrates come together during the tRNA-modification reaction considering that their respective binding sites are located about 20 angstrom away from each other. By re-investigating the available structures of Elp3 (or the Elp1-2-3 subcomplex) bound to tRNA, the authors discovered the existence of a tunnel that connects both active sites. Using Elp3 from the archaeon Methanocaldococcus infernus (MinElp3) they were able to reconstitute the tRNA modification reaction, and confirmed the role and relevance of this tunnel in transporting acetate between the active sites, using a combination of site-directed mutagenesis, enzymology and isotope incorporation experiments.

While we believe the results described in the paper are interesting for the Elp3 field, and the tRNA modification field in general, and could present a significant step forward in the understanding of the regulation of the Elp3-catalyzed tRNA modification reaction, in certain cases the conclusions that are made are insufficiently supported by strong experimental evidence, as detailed below. Additionally, to increase the importance of their findings for a broader audience it would be interesting to extrapolate the results to (human) Elongator and to discuss the potential implications for human disease.

Major Remarks

- The discovery of the tunnel is based on the cryo-EM structure of the yeast Elp1-Elp2-Elp3 complex bound to tRNA, and its existence is then extrapolated to (all) other Elp3-tRNA complexes - including MinElp3 - based on the conservation of the residues that align the tunnel. While I understand that it might not be possible to solve an experimental structure of a MinElp3-tRNA complex, did the authors try to model such a complex with AlphaFold 3 to assess whether the tunnel is also predicted in that case? Such a model would moreover also facilitate the design and interpretation of subsequent mutagenesis studies.

- Figure 1B: Can the authors comment on why the tRNA modification reaction is slower with acetate compared to AcCoA? Where do the authors hypothesize acetate enters the protein? Does it enter via the AcCoA pocket or would it be possible that it directly binds into the rSAM pocket, hence bypassing the tunnel?

- Related to the above remark: in Figure 2B the effect of the I450W mutation on tRNA modification is only tested with AcCoA. This should also be tested using acetate as substrate, to assess whether acetate needs to go through the tunnel or can directly access the rSAM pocket. This should also be done with additional mutants: see my remark below.

- To test the relevance of the tunnel for the tRNA modification reaction, the authors test one mutation (I450W). They verify that this mutation does not affect tRNA binding and AcCoA turnover, however, for completeness, they should also test whether this mutation affects SAM binding. Additionally, testing only one mutation in the tunnel, seems insufficient to strongly support the main conclusions of the manuscript, especially considering that I450 is not one of the completely conserved residues within the tunnel (see Fig. S3). To solidify their conclusions, other highly conserved (positively and negatively charged) residues in the tunnel should be mutated and tested: e.g. K150, E207, K266, H270, R440. Ideally, it would be recommended to use computational methods to verify that these mutations do not affect the folding of the protein.

- Figure 4 and associated conclusions. To prove that the loss of tRNA modification of the K302R, K302A, Y304F and Y304A mutations is really due to impaired binding of acetate rather than to other factors, a direct binding assay to measure binding of acetate to MinElp3 should be performed (for example ITC would be a very good option).

- In the discussion it would be interesting to add a paragraph to place the results in the context of human Elongator and the associated diseases. Does any of the known disease mutations affect residues that align the newly described tunnel?

Minor remarks

- Figure 1A: it would be clearer to show Elp1, Elp2 and Elp3 in different colors. This will allow to better assess whether or not Elp1 and Elp2 contribute to the formation of the tunnel.

- Figure 2D: The author conclude based on these data that I154W does not affect AcCoA hydrolysis. I do not fully agree with this statement. The data point to a nearly two-fold decrease in AcCoA hydrolysis. It is not because this effect does not reach statistical significance, that it is correct to state that there is no effect. Indeed, by including more data a statistically significant difference might still be obtained. The authors should at least state that there might be a difference, although no statistical significance is obtained.

Reviewer #3

(Remarks to the Author)
As the catalytic core of the Elongator complex, Elp3 executes essential enzymatic functions in tRNA modification processes. Previous investigations have postulated three distinct molecular mechanisms for Elp3-mediated tRNA modification. However, none of these hypotheses have been conclusively validated through definitive biochemical evidence from in vitro enzymatic assays demonstrating intrinsic catalytic activity.

This investigation employs a multidisciplinary approach combining structural analysis with isotope incorporation assays, also corroborate by in vitro enzymatic activity measurements using recombinant active minElp3, demonstrates that Elp3 alone can successfully catalyze U34 tRNA modification through a conserved channel, which spatially coordinates the radical S-adenosylmethionine (rSAM) domain with the acetyl-CoA (AcCoA) binding site. Then, the mechanistic model gains further support from systematic site-directed mutagenesis targeting key residues lining the substrate tunnel.

While these findings offer valuable mechanistic insights, we recognize that the current experimental validations have relied on the use of isolated minElp3. To more comprehensively replicate the physiological context, we strongly propose conducting additional validation via in vitro reconstitution of the complete Elongator complex derived from other organisms. Demonstrating equivalent catalytic activity with the fully assembled complex would not only render the conclusions more compelling but also more closely mimic the native biological conditions. This enhancement would significantly contribute to

the overall rigor and quality of the manuscript. In the absence of such validation, the presentation of their findings should be moderated to reflect the current limitations.

Overall, the logic of this study is clear and the evidence is sufficient. I recommend its publication in your journal after supplementing the aforementioned experiments.

1. Figure 1C, according to other experiments, the concentration of acetate is up to 2 or 10 mM. The author should repeat this assay by using high concentration of acetate.
2. Figure S8, the EMSA is not an idea assay to test binding between tRNA and Elp3. How about Isothermal Titration Calorimetry (ITC) or MicroScale Thermophoresis (MST)?
3. Figure S11, why the author did molecular docking using yeast Elp3 instead of minElp3?
4. Do the mutants depicted in Figure 4 exhibit similar tRNA binding capabilities or AcCoA hydrolysis activities as WT?

Version 1:

Reviewer comments:

Reviewer #1

(Remarks to the Author)
The authors adequately addressed reviewers' comments.

Reviewer #2

(Remarks to the Author)
I want to congratulate the authors for the very meticulous way in which they addressed all my concerns and remarks. They performed the majority of the requested additional experiments, or else convincingly argued why the proposed experiments were not justified. I appreciate the significant amount of extra effort this has taken, and I hope the authors agree that including those experiments closed some loose ends and significantly strengthened the conclusions.

I have no further comments and support the publication of the manuscript in its current form.

Reviewer #3

(Remarks to the Author)
I have carefully reviewed the authors’ point-by-point responses to my previous comments and other reviews' comments. Overall, I appreciate the additional experiments and clarifications provided, which have strengthened the manuscript. Some of my specific technical concerns have been addressed, either through new data or well-reasoned justifications. Importantly, the authors have carefully revised the language throughout the manuscript to accurately convey the findings within the context of the model system, while also offering testable hypotheses for the eukaryotic complex. The study now presents a robust and coherent mechanistic model for minElp3, with clear implications for the broader Elongator field.

Open Access This Peer Review File is licensed under a Creative Commons Attribution 4.0 International License, which permits use, sharing, adaptation, distribution and reproduction in any medium or format, as long as you give appropriate credit to the original author(s) and the source, provide a link to the Creative Commons license, and indicate if changes were made.
In cases where reviewers are anonymous, credit should be given to 'Anonymous Referee' and the source.
The images or other third party material in this Peer Review File are included in the article's Creative Commons license, unless indicated otherwise in a credit line to the material. If material is not included in the article's Creative Commons license and your intended use is not permitted by statutory regulation or exceeds the permitted use, you will need to obtain permission directly from the copyright holder.
To view a copy of this license, visit https://creativecommons.org/licenses/by/4.0/

REVISION SUMMARY

*We thank the reviewers and the editor for their helpful comments, questions, and suggestions! In this significantly revised version of the manuscript we have added substantial additional experiments and analysis including: modeling the Elp3 molecular tunnel in archaeal and eukaryotic systems using both available structures and AlphaFold predictions to show this feature is conserved (**Figure S3**); adding an additional tunnel-blocking mutation (M165W) with full biochemical characterization that further supports the functional role of the Elp3 molecular tunnel (**Figure 2**); significantly expanding the biochemical characterization (SAM binding, tRNA binding, AcCoA hydrolysis) of conserved, tunnel-lining residues (E207, K302, Y304) to show and characterize their functional importance (**Figure 4**); adding new MST-based SAM binding assays to show key tunnel-lining / active site mutations do not impact SAM binding (**Figure 4**); and adding acetate-based activity assays for tunnel-blocking mutations to clarify the substrate entry route of acetate (**Figure 2**).*

We have moderated interpretative language throughout to qualify claims related to hypothesized structure-level interactions and the conservation of mechanism between archaeal and eukaryotic systems. We have also added new discussion putting some of our mechanistic results in the context of eukaryotic Elongator and the potential disease relevance of tunnel-lining residues in humans.

Further details, many additional changes, and our point-by-point response to all reviewer comments are below.

REVIEWER COMMENTS

Reviewer #1 (Remarks to the Author):

Geissler et al. uncovered a novel catalytic mechanism of Elp3 forming Cm5U formation. Cm5U and its derivatives are widely conserved modification in eukaryotes and archaea. Lack of cm5U causes defects in cell fitness and involved in human diseases. Elp3 has two substrate binding sites, radical SAM motif and acetyl-CoA binding site. Previous reports showed that 5-dA radical generated at the radical SAM domain is activating acetyl group of acetyl-CoA, and then activated acetyl-CoA attack tRNA to form cm5U modification. However, since two reaction sites are far from each other, how 5-dA radical activates acetyl-CoA was outstanding question. In this manuscript, the authors conducted structural analysis using software that finds tunnels and channels in protein structure and found a molecular tunnel from Acetyl-CoA binding site to SAM binding site. Authors also discovered that cm5U formation is successfully conducted by acetate instead of acetyl-CoA. These observations support authors' proposed model that an acetate molecule released from acetyl-CoA travel through the molecular tunnel inside of Elp3 and activated at radical SAM domain and form the cm5U modification. The biochemical, mass spectrometric, and computational studies strongly support their reaction model. The manuscript is well written, experiments are appropriately designed and conducted, and their results robustly support authors' conclusion. The finding of unique catalytic mechanism underlying well-conserved and significant tRNA modification will be of great interest of the broad range of the readers of Nature Communications. This reviewer highly recommends publishing this paper.

We thank the reviewer for this excellent summary and highly supportive assessment!

One minor comment is that authors should discuss the conservation of tunnel structure among different Elp3 proteins. Authors found the tunnel in yeast Elp3 protein, but use the archaeal protein for the biochemical studies. I am wondering if the tunnel structure is also observed in archaeal homolog. If there is no available structure, checking a predicted structure of MinElp3 would be feasible to address this point

Yes, this is a great point, especially considering that we say the tunnel is conserved. There are no archaeal Elp3 structures with bound tRNA available, so we instead generated a MinElp3-tRNA anti-codon stem loop (ASL) complex with AlphaFold3 and carried out similar tunnel analyses with CAVER. Additionally, we also analyzed the presence of tunnels in both an AlphaFold3-generated mouse Elp3-tRNA ASL complex and in the experimentally determined human Elp3-tRNA complex (from Elongator-tRNA cryo-EM structure 8PTX). In all of these cases, as with the yeast structure shown in the main text of the manuscript, we find similar, continuous, enclosed molecular tunnels that connect the AcCoA binding site on the KAT domain to the rSAM active site. As would be expected, the details of the size and shape of these tunnels vary by a small amount across each of the structural models, depending on the exact positioning of tunnel-lining Elp3 sidechains and tRNA bases; but of course, in solution, these residues and bases will be at least somewhat dynamic, so the predicted tunnels are only a simple, static snapshot of the tunnel topology. Overall, from both AF3-generated and experimentally determined structures spanning archaeal, yeast, and mammalian Elp3-tRNA complexes we find strong evidence that the molecular tunnel is conserved across all domains of life.

*We have added a sentence to the main text of the manuscript in the first paragraph of the results section (**lines 123-126**) highlighting this point, and added the new Elp3-tRNA models from Min Elp3, mouse Elp3, and human Elp3, along with their CAVER-calculated tunnels, as the new **Figure S3**.*

Reviewer #2 (Remarks to the Author):

In their manuscript the authors present new insights into the Elp3-catalyzed tRNA modification reaction, through the discovery of a tunnel that is used to shuttle the reaction intermediate acetate from the acetyl-CoA binding pocket to the radical SAM active site. In eukaryotes, including humans, Elp3 is the catalytic subunit of a large multi-protein complex, called Elongator consisting of the proteins Elp1-6, which modifies tRNA wobble uridines to 5-carboxymethyluridine (cm5U). Mutations in Elp3 and other Elongator subunits are linked to various diseases, including cancer and neurodegenerative and neurodevelopmental diseases. However, in many archaea and some bacteria, Elp3 exists as a stand-alone enzyme that conducts the same reaction. Due to the challenges in reconstituting the Elp3-catalyzed tRNA modification reaction, and despite the availability of structures of Elp3 and Elongator subcomplexes, an open question in the field that remained is how the SAM and AcCoA substrates come together during the tRNA-modification reaction considering that their respective binding sites are located about 20 angstrom away from each other. By re-investigating the available structures of Elp3 (or the Elp1-2-3 subcomplex) bound to tRNA, the authors discovered the existence of a tunnel that connects both active sites. Using Elp3 from the archaeon Methanocaldococcus infernus (MinElp3) they were able to reconstitute the tRNA modification reaction, and confirmed the role and relevance of this tunnel in transporting acetate between the active sites, using a combination of site-directed mutagenesis, enzymology and isotope incorporation experiments.

While we believe the results described in the paper are interesting for the Elp3 field, and the tRNA modification field in general, and could present a significant step forward in the understanding of

the regulation of the Elp3-catalyzed tRNA modification reaction, in certain cases the conclusions that are made are insufficiently supported by strong experimental evidence, as detailed below. Additionally, to increase the importance of their findings for a broader audience it would be interesting to extrapolate the results to (human) Elongator and to discuss the potential implications for human disease.

We thank the reviewer for their excellent summary and recognition of the potential impacts and advances from our work. The reviewer raises important points about further support for certain conclusions, and we address each of these in detail below.

Major Remarks

- The discovery of the tunnel is based on the cryo-EM structure of the yeast Elp1-Elp2-Elp3 complex bound to tRNA, and its existence is then extrapolated to (all) other Elp3-tRNA complexes - including MinElp3 - based on the conservation of the residues that align the tunnel. While I understand that it might not be possible to solve an experimental structure of a MinElp3-tRNA complex, did the authors try to model such a complex with AlphaFold 3 to assess whether the tunnel is also predicted in that case? Such a model would moreover also facilitate the design and interpretation of subsequent mutagenesis studies.

*Yes, this is an important point, also raised by Reviewer 1. See our comments to Reviewer 1 above, but we have now analyzed AlphaFold3-generated or experimentally determined EM structures of Min (AF3), yeast (EM), mouse (AF3), and human (EM) Elp3-tRNA complexes with CAVER, and find similar enclosed molecular tunnels in all of these structures. This additional analysis has been added to the first paragraph of the Results section (**lines 123-126**), and the new predicted tunnels and analyzed Elp3-tRNA models have been added as the new **Figure S3**.*

- Figure 1B: Can the authors comment on why the tRNA modification reaction is slower with acetate compared to AcCoA? Where do the authors hypothesize acetate enters the protein? Does it enter via the AcCoA pocket or would it be possible that it directly binds into the rSAM pocket, hence bypassing the tunnel?

*As we had originally noted later in the manuscript (in the 'Conserved Elp3 rSAM active site…' results section), the effective local concentration of one molecule of acetate released into the molecular tunnel by AcCoA hydrolysis is calculated to be ~4.5 M, *significantly* higher than the ~mM reaction concentrations we are able to use in our radiolabel-based experiments. If acetate binding is very weak to the rSAM active site (which is supported by the lack of any observed saturation in tRNA modification activity levels up to 2 mM added acetate, see new Figure S7), it is likely that a smaller fraction of rSAM active sites have bound acetate under the reaction conditions where we add exogenous acetate at mM concentrations, compared to AcCoA-based reactivity where the local acetate concentration in the enclosed tunnel will be much higher. This would result in slower modification rates for reactions with exogenous acetate.*

*To make this point more clear, and relate it directly to the slower reaction observed for acetate, we moved up this discussion of the tunnel volume and local acetate concentration to the second results section (see **lines 176-186**).*

The second two questions from this point are answered in the point below.

- Related to the above remark: in Figure 2B the effect of the I450W mutation on tRNA modification is only tested with AcCoA. This should also be tested using acetate as substrate, to

assess whether acetate needs to go through the tunnel or can directly access the rSAM pocket. This should also be done with additional mutants: see my remark below.

*We added new experiments testing the tRNA modification activity of both I154W and the new M156W (see below) Elp3 tunnel-blocking mutants with acetate to **Figure 2B** (M156W) and **Figure S10,B** (I154W). For both of these tunnel-blocking mutants, we find that they are inactive with either AcCoA or acetate cofactors. This suggests that acetate cannot effectively access the rSAM active site directly and must pass through the tunnel and into the structured/tRNA-bound active site to successfully promote tRNA modification. We have added a sentence about this to the results section (**lines 221-224**).*

This model is again consistent with acetate being very weakly bound by the rSAM active site itself. For acetate to access the rSAM active site directly in the tunnel-blocked mutants I154W or M156W, acetate would need to bind the Elp3 rSAM active site when tRNA is unbound, and then stay associated with the active site while tRNA re-binds, in order to generate complexes that could undergo modification reactions. If acetate is very weakly bound to the rSAM site and rapidly dissociates, it is perhaps unsurprising that this coordinated two-step process (acetate binding followed by tRNA binding) is infeasible to produce tRNA modification reactivity in the tunnel-blocked mutants where acetate would need to access the rSAM site directly (via Elp3 with no tRNA bound).

- To test the relevance of the tunnel for the tRNA modification reaction, the authors test one mutation (I450W). They verify that this mutation does not affect tRNA binding and AcCoA turnover, however, for completeness, they should also test whether this mutation affects SAM binding.

*We developed a new MST-based assay to measure SAM binding to Elp3 under anaerobic conditions. We compared SAM binding in WT Elp3 versus tunnel-blocking mutants I154W and the added M156W (see below), and found that similar to the results for tRNA binding and AcCoA turnover, these mutants did not adversely affect SAM binding to Elp3. These results have been added to the results section (**lines 212-213**) and **Figure 2C** (for M156W) and **Figure S10,C** (for I154W).*

Additionally, testing only one mutation in the tunnel, seems insufficient to strongly support the main conclusions of the manuscript, especially considering that I450 is not one of the completely conserved residues within the tunnel (see Fig. S3). To solidify their conclusions, other highly conserved (positively and negatively charged) residues in the tunnel should be mutated and tested: e.g. K150, E207, K266, H270, R440.

It's not clear to us from this comment whether the reviewer is asking for: (1) additional tunnel-blocking mutations (ie mutating residues to Trp or similar) to specifically test how blocking or restricting the tunnel impacts Elp3 activity, or (2) mutations of conserved residues to Ala (or other charge altering mutations) that would test the overall importance of conserved residues found in the tunnel, but not necessarily directly assess tunnel function. We address and add new experimental data for both of these possibilities in turn:

*(1) **Additional tunnel-blocking mutations to directly assess the functional importance of the tunnel**. We chose additional, non-conserved tunnel-lining residues to mutate to Trp for added tunnel blocking experiments that will further support the main conclusion(s) of the manuscript. However, we first need to clarify here why it is critical to choose *non-conserved* residues for these experiments: the goal of the tunnel-blocking experiments is to show that restricting or*

*blocking the tunnel by making mutations that increase the size of tunnel-lining residues prevents tRNA modification activity by blocking the tunnel, and not by disrupting any other functions of the enzyme. By choosing *non-conserved* residues for these experiments, we maximize the chance that mutations at those positions do not disrupt other functions of the enzyme (eg tRNA binding, SAM binding, AcCoA hydrolysis). Conserved residues are of course much more likely to play a direct role in many of these other enzyme functions – indeed, many of the residues the reviewer suggests mutating are already known to impair Elp3 function for different reasons (e.g. K150 and K266 mutations have previously been shown to inactivate AcCoA hydrolysis – Lin et al, Nat. Comm. 2019). So mutations to those conserved tunnel-lining residues to *specifically test tunnel function* will not be useful, as they are known to disrupt enzyme function for other reasons. We apologize that this was not clear from the initial manuscript, and have added a sentence to the 'Blocking the molecular tunnel' results section (**lines 202-204**) to explain why we are specifically choosing non-conserved residues in these tunnel-blocking mutation experiments.*

*With this in mind, we chose two additional non-conserved tunnel-lining residues, M156 and V80, to mutate to Trp for additional tunnel-blocking experiments and full biochemical characterization. We expressed, purified, and reconstituted the FeS cluster for these new mutant Elp3 constructs. V80W was found to completely inactivate AcCoA hydrolysis activity, so we abandoned this mutation for tunnel-blocking experiments or further characterization (we also note that this highlights how even when choosing non-conserved residues, it is very difficult to find mutations that will block the tunnel without affecting any other functions of Elp3!). M156W however, was found to inactivate Elp3 tRNA modification activity (with either 14C-AcCoA or 14C-acetate), but have no significant effects on tRNA binding, SAM binding, or AcCoA hydrolysis activity compared to WT Elp3. In our structural models, M156W appears to more completely block the molecular tunnel, so we chose to move this data to **Figure 2** in the main text, and the previous I154W data to the Supporting Info in **Figure S10**. Together, we think that the added M156W mutation and accompanying full biochemical characterization data (tRNA binding, SAM binding, AcCoA turnover), in addition to the I154W data (for which we added SAM binding data), strongly support our hypothesis that mutations that block/restrict the molecular tunnel inactivate Elp3 by preventing acetate transport.*

*(2) **Mutations of highly conserved tunnel-lining residues in Elp3**. In this version of the manuscript, we have added full biochemical characterization (added assays for: tRNA binding, SAM binding, AcCoA hydrolysis, in addition to AcCoA- and acetate-based activity assays) of highly conserved, tunnel-lining rSAM residue mutations E207A, K302A, and Y304A (data added to **Figure 4**). In these experiments, we find that K302A completely abolishes tRNA modification activity (with AcCoA or acetate), has no significant effects on tRNA binding or SAM binding, but decreases AcCoA hydrolysis by ~3-fold compared to WT Elp3. Y304A completely abolishes tRNA modification activity (with AcCoA or acetate), results in a small (1.5-fold) decrease in tRNA binding affinity, and has no effect on SAM binding or AcCoA hydrolysis. In the case of E207A, we find this mutation likewise abolishes activity (with AcCoA or acetate), but has no significant impacts on tRNA binding, SAM binding, or AcCoA hydrolysis.*

These data clearly show that these highly conserved, in some cases charged, tunnel-lining residues are functionally important for tRNA modification. We argue that these residues, located in the active site within the tunnel, likely play roles in coordinating acetate within the tunnel; for more on these residues, see our related replies below to comments on 'Figure 4 and associated conclusions'.

More broadly, if the reviewer is asking how changes in overall tunnel charge, hydrophobicity, polarity, etc may affect acetate transport and tRNA modification activity – we think these are

interesting questions, but will likely require extensive, systematic mutagenesis of many tunnel-lining residues and their in-depth biochemical characterization, likely coupled to structural and/or molecular dynamics studies. We save this level of in-depth analysis of tunnel properties, which falls outside the scope of hypotheses/claims/conclusions in this manuscript, for future work. The combination of (1) tunnel-blocking mutations (M156W, I154W) that disrupt activity without disrupting other functions of Elp3 and (2) mutation of highly conserved tunnel-lining residues (E207A, K302A, Y304A) that show the clear functional importance of these residues, strongly support our conclusion that the molecular tunnel is important for Elp3 function.

*Relatedly, we also realized that the selected tunnel-lining residues originally shown in the sequence logo in the old Figure S3 (now **Figure S4**) were too conservative, and we have updated this figure to a version that has a fuller catalog of Elp3 residues whose sidechains compose part of the tunnel surface.*

Ideally, it would be recommended to use computational methods to verify that these mutations do not affect the folding of the protein.

*We used AlphaFold3 to predict the structures of WT vs I154W, M156W, E207A, K302A, or Y304A Elp3, and found that none of these mutations affect the predicted fold of the enzyme. The aligned AF3-predicted structural models for these Elp3 constructs have been added as **Figure S11** (I154W and M156W) and **Figure S17** (E207A, K302A, Y304A) and we have added reference to these predicted models in the results section (**lines 216-218**; lines **318-319**).*

- Figure 4 and associated conclusions. To prove that the loss of tRNA modification of the K302R, K302A, Y304F and Y304A mutations is really due to impaired binding of acetate rather than to other factors, a direct binding assay to measure binding of acetate to MinElp3 should be performed (for example ITC would be a very good option).

We agree that an acetate binding assay would be useful here to further support the proposed hypothesis that acetate directly interacts with these residues. We explored using ITC for this assay, but in discussions with biophysical core director who has extensive experience in ITC, found this technique to be ill-suited to measure acetate binding in this particular system because: (a) it is difficult to carry out rigorously air-free ITC experiments with the setup available to us (which would be required to maintain the 4Fe-4S cluster) and (b) we would need to titrate very high concentrations of charged acetate into high salt buffer solutions of Elp3/tRNA, resulting in huge heats of ionization when the acetate mixes with other ions in solution – this will be very difficult to deconvolve and separate out the much smaller heat contribution of the acetate-Elp3 interaction.

Instead, we carried out extensive attempts to develop acetate binding assays using either NMR saturation transfer difference (STD) or microscale thermophoresis (MST) experiments. Both of these techniques allow for rigorously air-free setup (with either sealed NMR tubes or MST capillaries prepared in a glovebox) and measurement. In the case of STD NMR experiments, we titrated increasing concentrations of acetate (up to 20 mM) into solutions of Elp3 + tRNA, and monitored transfer of magnetization from Elp3 to acetate over the course of the experiment. Here, we saw only weak, non-saturable signal increases, suggesting acetate binding is very weak and that non-specific binding effects dominate in this experimental setup where we are adding large amounts of acetate to bulk solution (rather than it being directly formed and sequestered within the Elp3 tunnel, as would happen with AcCoA-based reactivity within the enzyme); non-specific binding effects are a well-known limitation of STD NMR at high (~mM) ligand concentrations.

In the case of MST experiments, we titrated acetate into solutions of Elp3 + tRNA and a polyhistidine-binding fluorescent dye, and monitored the resulting change in relative fluorescence. While we developed similar experiments that worked well for quantifying SAM binding to Elp3 (as discussed above), we saw no change in MST signal even when titrating acetate concentrations up to 20 mM. This suggests that acetate binding to Elp3 is either extremely weak and/or acetate binding does not result in sufficient Elp3/dye conformational changes that can be detected in the MST experiment.

We now have multiple lines of evidence that suggest, unsurprisingly, that acetate binding to the rSAM site on Elp3 is very weak, making development of binding assays to quantify this interaction very challenging.

Although we were unable to develop an assay to directly measure acetate binding to the Elp3 active site, to help mitigate concerns that mutations to active site / tunnel residues E207, K302, and Y304 could be disrupting other functions besides acetate binding, we used MST-based assays to measure SAM binding to Elp3 for WT versus these active site mutations. Here, we found that E207A, K302A, and Y304A Elp3 mutations all had SAM binding affinity similar to WT, suggesting that these mutations do not significantly disrupt SAM binding and the rSAM active site structure. Furthermore, we also added experiments to measure tRNA binding and AcCoA hydrolysis to all 3 of these mutants versus WT. Here, we find that K302A and Y304A mutations do have some moderate impacts on tRNA binding (Y304A decreases tRNA binding by ~1.5-fold) or AcCoA hydrolysis (K302A decreases AcCoA hydrolysis by ~3-fold), but E207A has WT-level tRNA binding and AcCoA hydrolysis. We have added all of this new functional data to ***Figure 4****, and argue that this together suggests that E207 may play a key role in coordinating/positioning acetate, since the E207A disrupts activity entirely, but has no effects on tRNA binding, SAM binding, or AcCoA hydrolysis. We suggest the possibility that the roles of K302 and Y304 may be multifunctional, since their Ala mutations completely abolish activity, but have only moderate effects on tRNA binding or AcCoA hydrolysis. All of this has been added and discussed in the last section of the results (****lines 360-382****).*

We have also moderated the language in this section, and tried to make it very clear that these are proposed/hypothesized roles for the identified residues, and that it is likely high-resolution structures with acetate and/or at different reaction stages may be required in the future to unambiguously assign the atomic-level roles of individual residues during the reaction cycle (namely, ***lines 380-382****, but we have also softened language throughout this section).*

- In the discussion it would be interesting to add a paragraph to place the results in the context of human Elongator and the associated diseases. Does any of the known disease mutations affect residues that align the newly described tunnel?

*Yes, interestingly some cancer-related mutations identified in tumor sequencing databases do line the molecular tunnel. We note that these are somatic mutations identified in tumors, but require future characterization to understand if/how they are functionally significant in cancer. We have added a paragraph at the end of the discussion (****lines 513-526****) describing this, and added a figure to the supporting information (****Figure S19****) showing the location of these tunnel-lining, cancer-associated mutations in human Elp3. Thank you for this suggestion!*

Minor remarks

- Figure 1A: it would be clearer to show Elp1, Elp2 and Elp3 in different colors. This will allow to better assess whether or not Elp1 and Elp2 contribute to the formation of the tunnel.

OK, we have changed the Elp1 (gray) and 2 (tan) colors to make these more distinct, and updated ***Figure 1A****.*

- Figure 2D: The author conclude based on these data that I154W does not affect AcCoA hydrolysis. I do not fully agree with this statement. The data point to a nearly two-fold decrease in AcCoA hydrolysis. It is not because this effect does not reach statistical significance, that it is correct to state that there is no effect. Indeed, by including more data a statistically significant difference might still be obtained. The authors should at least state that there might be a difference, although no statistical significance is obtained.

OK, yes, this is a fair point. The new tunnel-blocking mutation we added in this version of the manuscript – M156W – appears to block the tunnel more fully in our mutagenesis models, and so we've opted to have the M156W model and data in ***Figure 2****, and have moved the I154W data to the Supplement (****Figure S10****). In the updated legend for the* ***Figure S10*** *I154W data, to address this point about the changes and significance of the AcCoA hydrolysis data, we now note: '****Elp3 I154W (yellow) may moderately reduce AcCoA hydrolysis activity in the presence of substrate tRNA Arg^{UCU} compared to WT Elp3 (teal), but the measured differences were not statistically significant****' (****lines 171-173*** *in the Supplement).*

Reviewer #3 (Remarks to the Author):

As the catalytic core of the Elongator complex, Elp3 executes essential enzymatic functions in tRNA modification processes. Previous investigations have postulated three distinct molecular mechanisms for Elp3-mediated tRNA modification. However, none of these hypotheses have been conclusively validated through definitive biochemical evidence from in vitro enzymatic assays demonstrating intrinsic catalytic activity.

This investigation employs a multidisciplinary approach combining structural analysis with isotope incorporation assays, also corroborate by in vitro enzymatic activity measurements using recombinant active minElp3, demonstrates that Elp3 alone can successfully catalyze U34 tRNA modification through a conserved channel, which spatially coordinates the radical S-adenosylmethionine (rSAM) domain with the acetyl-CoA (AcCoA) binding site. Then, the mechanistic model gains further support from systematic site-directed mutagenesis targeting key residues lining the substrate tunnel.

While these findings offer valuable mechanistic insights, we recognize that the current experimental validations have relied on the use of isolated minElp3. To more comprehensively replicate the physiological context, we strongly propose conducting additional validation via in vitro reconstitution of the complete Elongator complex derived from other organisms. Demonstrating equivalent catalytic activity with the fully assembled complex would not only render the conclusions more compelling but also more closely mimic the native biological conditions. This enhancement would significantly contribute to the overall rigor and quality of the manuscript. In the absence of such validation, the presentation of their findings should be moderated to reflect the current limitations.

Overall, the logic of this study is clear and the evidence is sufficient. I recommend its publication in your journal after supplementing the aforementioned experiments.

We thank the reviewer for this summary that recognizes the potential impacts of this work, and the strength of evidence presented for the model Min Elp3 system. To address the broader point about testing all of these hypotheses with purified, reconstituted eukaryotic Elongator complex: we note that tRNA modifying activity of the twelve protein, ~900 kDa Elongator complex has never been successfully reconstituted in vitro. While we agree this could help support some of our observations from the model Min Elp3 system, reconstitution of Elongator in vitro activity remains a large and long-term challenge for the field. In the same way that tractable, bacterial homologs have long served as suitable model systems for understanding conserved enzyme mechanisms, we believe that the high conservation of Elp3 sequence and structure (and tunnel topology, as discussed above) observed between archaeal and eukaryotic Elp3s allows us to probe and characterize conserved mechanistic principles in the model, biochemically tractable Min Elp3 system. We take the reviewer's suggestion to tackle the major, unsolved problem of Elongator in vitro activity reconstitution as an important challenge for future work!

*We have added a paragraph at the end of the discussion (**lines 513-518**) describing why we think our data from the model archaeal Elp3 likely also applies in the case of Elongator (ie conservation of key residues and the molecular tunnel feature; tunnel-lining residues that have been shown to also be important in Elongator). We have also moderated the language related to conservation of mechanism throughout the paper, so that it is hopefully clear to readers that our data supports a functional molecular tunnel and acetate transport in archaeal Elp3, and we propose/hypothesize that this is likely the case in eukaryotic Elp3/Elongator as well (but do not directly show this).*

1. Figure 1C, according to other experiments, the concentration of acetate is up to 2 or 10 mM. The author should repeat this assay by using high concentration of acetate.

*We repeated a very similar assay with acetate concentrations up to 2.2 mM: we monitored, in triplicate, the relative amounts of 14C-tRNA product formed after 2 h in Elp3 reactions with acetate concentrations ranging from 0.07 – 2.2 mM, and plotted this activity versus acetate concentration (added as new **Figure S7**, and described in the main text at **lines 184-186**). This nicely shows that activity increases linearly with added acetate to concentrations above 2 mM acetate; the lack of any saturation in activity levels through 2 mM acetate concentration is consistent with very weak binding of acetate itself to the rSAM active site.*

2. Figure S8, the EMSA is not an idea assay to test binding between tRNA and Elp3. How about Isothermal Titration Calorimetry (ITC) or MicroScale Thermophoresis (MST)?

In the case of relatively tight Elp3-tRNA complexes (~ hundred nM Kds), EMSA should be a suitable and robust technique to measure relative tRNA binding affinities. Indeed, EMSAs are a well-established technique to measure RNA-protein interactions (Fillebeen et al, J Vis Exp 2014, doi: 10.3791/52230; Perea et al, Anal Biochem 2020, doi: 10.1016/j.ab.2020.113736; and many others), and other labs have extensively used EMSAs to measure Elp3-tRNA or Elongator-tRNA interactions previously (for example: Glatt et al, Nat Struct Mol Biol 2017, doi: 10.1038/nsmb.3265; Chramiec-Glabik et al, Protocol 2023, doi: 10.1007/978-1-0716-3191-1_3). In our binding experiments, we saw clear, concentration-dependent gel shifts that cleanly differentiated unbound tRNA from Elp3-tRNA complex. For these reasons we used EMSAs to monitor Elp3-tRNA binding, and expect that the relative changes in observed binding affinities are well-determined.

3. Figure S11, why the author did molecular docking using yeast Elp3 instead of minElp3?

*For **Figure S15** in the updated manuscript: because there is no experimental structure available for MinElp3 with bound [4Fe-4S] cluster, 5'-dA, or tRNA, we chose to use the yeast Elp3-tRNA EM structure, which has all of these key features, for molecular docking of acetate into the Elp3 active site and tunnel. We reasoned that the presence of the [4Fe-4S] cluster, 5'-dA, and tRNA, are all likely necessary for a well-structured tunnel and rSAM active site, which would be a critical starting point for molecular docking. While we could generate MinElp3-tRNA complexes with AlphaFold, these models would still lack the FeS cluster and 5'-dA, so we chose the experimentally determined yeast Elp3-tRNA structure as the best option.*

4. Do the mutants depicted in Figure 4 exhibit similar tRNA binding capabilities or AcCoA hydrolysis activities as WT?

*Thank you for this important suggestion. In the updated manuscript, we have added assays and new data that directly measure SAM binding, tRNA binding, and AcCoA hydrolysis for all of the mutants in **Figure 4**. We have added this data to **Figure 4C-E**, and find that: (1) none of the mutations effect SAM binding to the rSAM active site; (2) Y304A has a small effect on tRNA binding (decrease by 1.5-fold); and K302A has a moderate effect on AcCoA hydrolysis (decrease by ~3-fold). In light of these added experiments, we have changed our language in this section of the results, to suggest that K302 and Y304 may be multifunctional residues – they are essential for activity, playing a role in tRNA binding or AcCoA hydrolysis, but may also help bind/position acetate. E207, which has not been functionally characterized in any Elp3 or Elongator system, does not seem to play any role in SAM binding, tRNA binding, or AcCoA hydrolysis, and so we suggest it's importance for tRNA modification activity may result from it helping with acetate binding/positioning.*

*However, we have also moderated the language in this section to make it clear that these are **proposed** roles for these residues, and that capturing acetate-bound Elp3 structures during different reaction states, may be required in the future to unambiguously assign the functional and atomic-level roles for these active site residues (a significant challenge for the future!)*
